# Red flags in global autism data: a forensic analysis of prevalence patterns and official aid dependencies

**DOI:** 10.3389/fpsyt.2025.1575940

**Published:** 2025-10-15

**Authors:** Jun Qiu, Alishba Hania

**Affiliations:** College of Education and Humanities, Nanchang Institute of Technology, Nanchang, China

**Keywords:** autism spectrum disorder, incidence, panel data, financial aid, Benford law

## Abstract

**Introduction:**

The literature extensively examines the global incidence rate of autism, emphasizing the need to scrutinize reported figures for potential anomalies, particularly addressing overdiagnosis concerns.

**Methods:**

Our forensic analysis employing Benford's Law and Mean Absolute Deviation indicates significant statistical irregularities and potential overdiagnosis, especially post-DSM-5 implementation, suggesting diagnostic criteria changes drive upward trends. The segmented analysis reveals this relationship intensified in low-income countries post-DSM-5 while remaining non-significant in high-income nations.

**Results:**

Based on 206 countries over 1990-2019, our findings suggest official aid received causes upward trends in autism cases for both genders. Sub-sample analysis indicates positive effects are pronounced in countries with low income, health expenditures, mental health services, government effectiveness, and weak democracies. Results remain robust through instrumental variable and lagged analyses addressing endogeneity concerns.

**Discussion:**

While Benford's Law suggests overdiagnosis patterns, both genuine increases and diagnostic inflation produce similar empirical results, preventing definitive conclusions. Nevertheless, these statistical red flags warrant future research and governmental vigilance when monitoring dramatic prevalence increases. This research addresses a critical literature gap, encouraging scholarly inquiry into reported autism prevalence complexities.

## Key message

Global autism diagnosis rates are potentially distorted by international aid and diagnostic criteria changes, particularly in low-income countries. Our study using Benford’s Law reveals complex interactions between financial support, healthcare systems, and medical reporting that challenge conventional understanding of autism prevalence.

## 1 Introduction

The Signal Detection Theory, a generally applied framework in psychology and epidemiology, highlights the significance of discriminating genuine signals from background noise (1). In the context of autism spectrum disorder prevalence, the term ‘signal’ implies the authentic rise in cases, while ‘noise’ comprehends probable distortions or misreporting. This theoretical framework becomes particularly relevant when examining global autism prevalence patterns, as failure to address anomalies poses the risk of misconstruing authentic epidemiological trends or, conversely, neglecting systemic issues within reported data and diagnostic processes. The historical evolution and prevalence of autism spectrum disorder exemplify this challenge, having undergone significant shifts across different parts of the world that reflect changes in diagnostic criteria, awareness, and societal discernments. Initially identified in the mid-20th century, autism was considered rare, and its prevalence was underestimated due to limited awareness and diagnostic tools. However, contemporary examination of global incidence rates reveals the need to not only assess the extent of reported increases but also systematically examine these figures for probable anomalies that may confound our understanding of true epidemiological patterns.

Over time, advancements in research and refinements in diagnostic criteria, such as the introduction of the DSM-III, DSM-IV, and DSM-5 (2–4), contributed to an apparent surge in reported autism cases. Theories including the broadening of the autism spectrum and greater awareness leading to more precise diagnoses have been proposed to explain this rise (5, 6). However, awareness alone cannot fully account for the increase, as it overlooks additional contributory factors (7). Recent theoretical frameworks, particularly the prevalence inflation hypothesis, argue that mental health awareness campaigns may inadvertently inflate reporting by both improving recognition of genuine cases and encouraging overinterpretation of subclinical symptoms (5, 8, 9). Complicating matters further, potential diagnostic overlap between autism spectrum disorder and personality disorders, given shared features in social communication, emotional regulation, and interpersonal functioning, can result in misattribution and diagnostic uncertainty (10).

These complexities are intensified by systemic incentives, as individuals with ASD diagnoses often gain preferential access to services and support compared to those with other developmental conditions. For instance, the Israel Ministry of Health in 2008 announced that children with ASD are eligible for an extended basket of care (14 hours weekly), additional treatments, and special education facilities from their first year of life through the age of 7, with added treatments available for older children and adolescents up to the age of 18 (11). Conversely, children identified with other developmental or mental disorders often do not have access to these extensive facilities. Therefore, the convergence of shared symptomology, increased awareness, destigmatization, and special ASD-specific services may create inducements for the overdiagnosis of ASD in comparison to other conditions (12).

**Figure 1:**
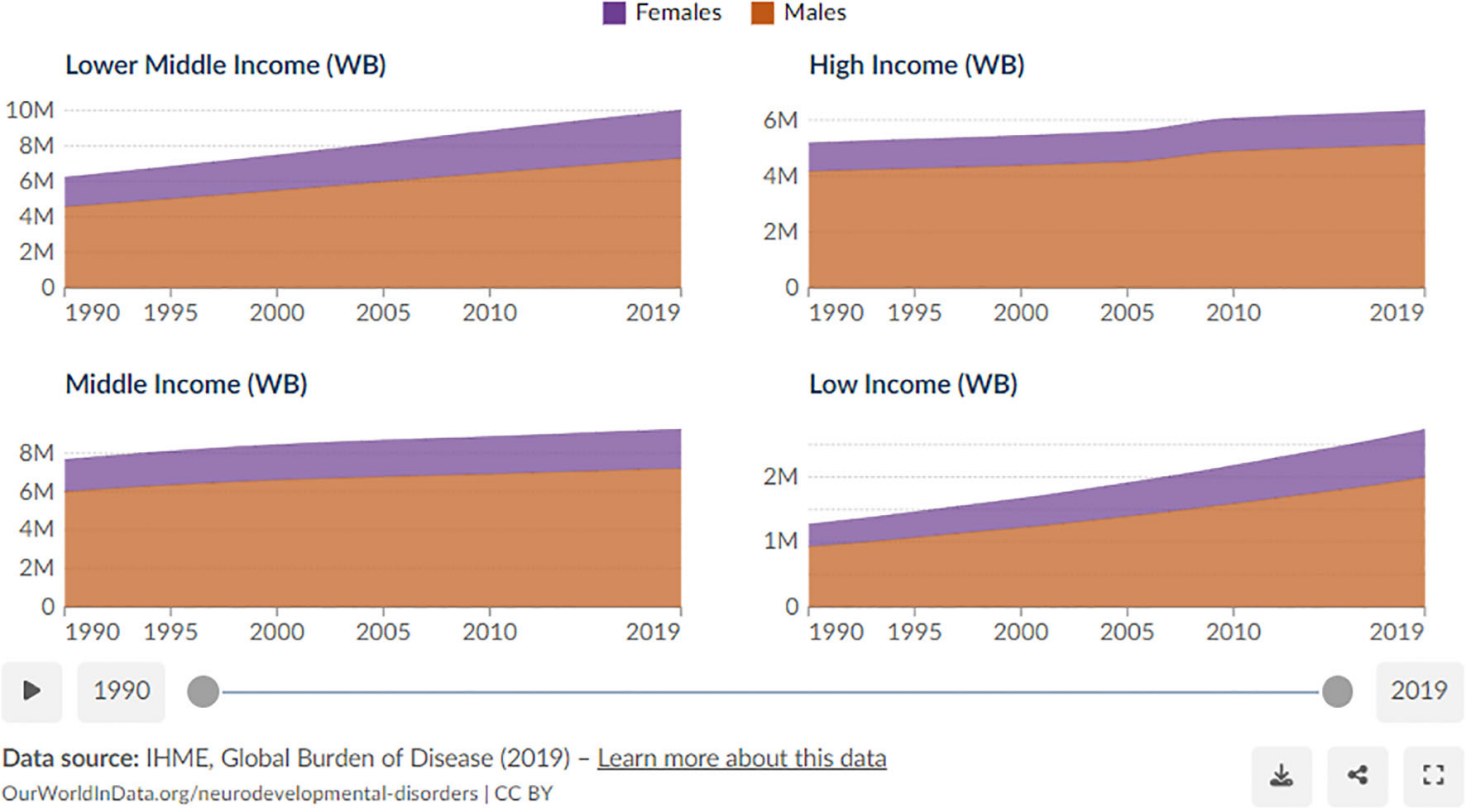
Gender-based statistics of autism cases. Data supported by the institute for health metrics and evaluation (IHME).

Data from the Global Burden of Disease Study (13) reflect these trends, revealing that high-income countries exhibited only a modest 20 percent relative increase in autism incidence between 1990 and 2019 (14), whereas low-income countries experienced a striking 114 percent absolute increase, with diagnosed cases rising from 344,606 in 1990 to 736,655 in 2019 (**Figure 1**). This disparity underscores the importance of examining contextual influences such as healthcare infrastructure, diagnostic capacity, and awareness, since understanding these dynamics is vital not only for identifying potential anomalies in reporting but also for shaping future research directions in the field (15). Researchers have consistently emphasized the inadequate healthcare resources in low-income countries, suggesting that these nations may face challenges in identifying and addressing mental health issues such as ASD (16–19).

Nevertheless, a closer inspection of secondary data spanning from 1990 to 2019 discloses a surprising inclination. Contrary to expectations (20, 21), there is a consistent and even elevated growth of autism cases in low-income countries, surpassing the rates observed in high-income countries. This unforeseen rise prompts an exploration of potential influencers that underwrite this phenomenon. In a study conducted by Davidovitch et al. (22), clinicians expressed concerns regarding the perceived overdiagnosis of both Autism Spectrum Disorder (ASD) and Attention-Deficit/Hyperactivity Disorder (ADHD). Consequently, one plausible explanation is the heightened emphasis on the disorder might have led to overdiagnosis, contributing to the observed growth. Furthermore, researchers highlight the overlap in symptoms between ASD and some other conditions, including intellectual and learning disabilities, as well as developmental language disorders (23–25). These diagnostic challenges are compounded by significant limitations in epidemiological surveillance systems, particularly age-restricted data collection that focuses primarily on eight-year-old populations while providing limited information on autism prevalence during adolescence, youth, or adulthood (26). Additionally, substantial gender disparities in diagnostic practices contribute to systematic underestimation, as traditional diagnostic criteria and assessment tools may inadequately capture autism presentations in females, leading to potential underdiagnosis in this population and skewing overall prevalence estimates toward male-dominated samples.

To systematically detect potential reporting anomalies and overdiagnosis patterns, we employ Benford’s Law, which has proven effective in healthcare fraud detection (27–29) and identifying fabricated data in scientific research that subsequently led to retractions (30). The law is particularly suited for autism prevalence data as it naturally spans multiple orders of magnitude across countries, a key requirement for Benford’s Law applicability (31), and can detect systematic reporting bias when external incentives influence diagnostic patterns (32). Probing deeper into this proposition, we examined the correlation between the growth pattern of autism cases and the influx of health-related aid, which exposed a significant connection. This nuanced analysis sheds light on the interplay of factors prompting the identification and reporting of autism cases on a global scale. Our contribution centers around a comprehensive inspection of reported autism cases across 206 countries from 1990 to 2019, marking the first wide-ranging analysis for potential anomalies and overdiagnosis in this extensive panel data approach. Leveraging statistical methodologies such as Benford’s Law (28) and Mean Absolute Deviation (MAD) (33), we systematically examined the dataset to reveal previously unexplored insights. Building upon previous research that underscores the overdiagnosis of autism spectrum disorder (ASD) due to shared symptomology, heightened awareness, and exclusive ASD-specific services (22, 34–39), we extend this argument through a unique comparative lens.

Current research explored the association between net funding received for healthcare by countries and reported autism cases, with a precise focus on gender-specific data. To strengthen the rigor of our study, we introduced vital control variables, including maternal/neonatal disorders, mental health policy, government effectiveness, out-of-pocket expenditures, population size, sex ratio at birth, and gross domestic product. The breakdown of our data based on mental health service levels, income, health expenditure, and government effectiveness adds granularity to our findings. Ensuring the robustness of our results, we conducted thorough analyses across various subsamples, employed instrumental variable techniques with carefully justified lagged aid instruments that address bidirectional causality concerns through established economic theory, and explored different lagged selections with comprehensive diagnostic testing to verify instrument validity. This comprehensive approach strengthens the validity of our insights and contributes valuable information for policymakers, healthcare practitioners, and researchers aiming to enhance the accuracy of autism diagnoses globally. This strategic research not only provides a deeper understanding of the upsurge in autism cases but also establishes a robust foundation for weighing the reliability and validity of the reported statistics.

## 2 Literature and theoretical foundation

### 2.1 Autism prevalence and history of diagnosis in developmental disorders

Over the past century, the understanding and prevalence of autism spectrum disorder (ASD) have endured a significant revolution. Initially thought of as a rare condition with inadequate recognition, the term “autism” was devised by Leo Kanner in 1943 (40). As diagnostic criteria evolved, awareness improved, societal understanding enhanced, and reported prevalence rates surged from the 1990s to the early 2000s. Recent research has revealed the intricate interplay of genetic, environmental, and epigenetic aspects contributing to ASD development, with developments in genetics shedding light on associated risk genes (41, 42). Existing prevalence approximations, exemplified by the Centers for Disease Control and Prevention (CDC) reporting 1 in 54 children with ASD in the United States as of 2021 (43), reflect not only an increased actual incidence but also heightened awareness and sophisticated diagnostic criteria. This change in perception from scarcity to a more nuanced understanding highlights the complex nature of autism’s prevalence. The steady rise in Autism Spectrum Disorder (ASD) prevalence stands out among some severe developmental disorders (44). While a genetic source is well-established, with numerous probable contributing genes identified (45), it’s notable that hereditary and *de novo* gene variations account for only 10–20% of ASD cases (46). Current studies link ASD risk to various issues, including advanced parental age, maternal pregnancy-related factors, birth complications, the use of technological devices in early childhood and environmental effects (47; 48–51).

Despite these factors, ASD lacks biological indicators, trusting solely on developmental history assessment, clinical observations, questionnaires, and semi-structured interviews for diagnosis. Some researchers suggest that factors related to seeking a diagnosis and the diagnostic procedure itself underwrite the rising ASD trend. These comprise diagnostic expansion, the presence of other neurodevelopmental disorders, diagnostic substitution, coupling to services, awareness, resolution, and overdiagnosis (15, 52–56). The application of Benford’s Law to medical diagnostic data has emerged as a valuable forensic tool for detecting potential anomalies in healthcare reporting (57). Studies have successfully applied Benford’s Law to identify irregularities in clinical trial data, pharmaceutical research, and epidemiological surveillance systems, demonstrating its effectiveness in healthcare contexts where systematic reporting bias may occur (30, 58). Particularly relevant to autism prevalence analysis, Benford’s Law has proven to be able to detect fabricated or manipulated data in scientific research that subsequently led to journal retractions, indicating its utility for identifying anomalous patterns in medical datasets that span multiple orders of magnitude across different populations (28, 59).

**Figure 2:**
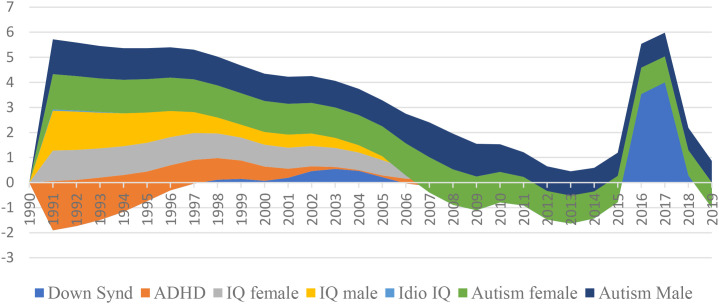
Growth rate of neurological disorders from 1990-2019.

Behavioral psychologists are known to come across challenges in differentiating between ASD and other developmental disorders owing to overlapping behavioral features. Down Syndrome, with its shared challenges in terms of communication and social interaction, might get misdiagnosed as ASD, especially during periods of decreasing prevalence (60, 61). Likewise, ADHD, with symptoms similar to impulsivity, hyperactivity, and inattention, could lead to overlaps in clinical assessments and probable misdiagnoses (62, 63). **Figure 2**, shows a comprehensive inspection of the growth of numerous developmental disorders and intelligence quotient (IQ) measured globally from years 1990 to 2019. Notably, this representation captures dynamic trends in Down Syndrome, Attention Deficit Hyperactivity Disorder (ADHD), and autism spectrum disorder (ASD), alongside male and female IQ scores. The latter part of the figure shows a significant spike in the co-occurrence of developmental disorders in 2016 and 2017, as demonstrated by substantial positive values in Down Syndrome, ADHD, and IQ measures. This period proposes a potential surge in the simultaneous growth of these conditions and there exists a potential for misdiagnosis, particularly concerning ASD.

Challenges arise from the lack of a standard definition and complications in quantifying overdiagnosis, complicating research, policy, and communication (64–66). In response to the challenges posed by the lack of a standard definition and difficulties in quantifying overdiagnosis in ASD, our research takes a comprehensive method. We employ a panel data methodology, analyzing reported ASD cases globally from 1990 to 2019. This technique allows us to report the intricacies of the growing prevalence while considering several factors that may influence the accuracy of diagnoses. To ensure a robust analysis, we meticulously control for possible influences from coexisting factors, including maternal/neonatal disorders, mental health policy, government effectiveness, out-of-pocket expenditures, population size, sex ratio at birth, and gross domestic product. By integrating these variables into our study, we aim to illuminate the nuanced interplay between these factors and the reported upsurge in ASD cases. This holistic approach enables a more comprehensive understanding of the dynamics contributing to the growing landscape of ASD prevalence.

### 2.2 Healthcare funding and ASD incidence

Contrary to the idea, “Giving some help to many in need is preferable to giving only some people a lot of help”, the situation for children with autism in developing countries is different. Given the novelty of the disorder and its lack of widespread understanding, families often find themselves caught off guard. In the pursuit of assistance, parents readily turn to specialized centers and institutes that place children in devoted care programs from the outset (67, 68). While early challenges could be managed by general specialists working with various developmental disorders (69), parents often incur additional expenses for exclusive care prompted by diagnoses from behavioral practitioners (70, 71). While wealthier families can pay for private practitioners, those with scarce resources may even incur debt in their desperation to support their children. Research in low-income countries (72–74) frequently noted the inclination to establish distinct services for autism assessment and interventions.

Despite effective lobbying for specialist provisions in affluent countries, these services are derived at a high cost. This not only poses financial loads but also hinders access to help for children with other developmental difficulties, resulting in waiting lists and a concentration of services in specific urban areas, leaving rural communities unsupported (75, 76). As special institutions allocate significant resources to accommodate autism cases, accompanied by substantial financial contributions from parents, there exists a potential for misdiagnosis/overdiagnosis (77, 78). This pattern of resource concentration and potential overdiagnosis has been documented across various contexts, yet existing research examining these dynamics has several critical limitations. Previous investigations (77, 78) have primarily relied on cross-sectional designs in high-income settings, failing to establish temporal relationships between funding patterns and diagnostic changes in developing countries where infrastructure is rapidly expanding through international aid. While earlier studies have observed these phenomena, they lacked systematic examination of the underlying economic mechanisms that drive such patterns, particularly how targeted mental health aid creates specific institutional incentives in resource-constrained environments.

Moreover, the theoretical mechanism connecting healthcare funding to diagnostic behavior works through multiple interconnected pathways that align with supplier-induced demand theory in health economics (79). Healthcare funding, mainly targeted aid for mental health services, creates systematic incentives that can influence diagnostic patterns through three primary channels: infrastructure development, professional training, and service expansion (80, 81). Firstly, increased funding leads to the establishment of specialized diagnostic facilities and recruitment of trained professionals, creating institutional capacity that must be utilized to justify continued investment. Second, funding-dependent training programs may inadvertently emphasize autism recognition over differential diagnosis, leading to diagnostic confirmation bias where practitioners are more likely to identify autism symptoms rather than alternative explanations (82–84). Third, it is proposed that the availability of autism-specific services funded through targeted aid creates systematic incentives for autism diagnoses over other developmental conditions that lack equivalent funding streams, as families and practitioners navigate toward better-resourced diagnostic categories (11, 84–86). This supplier-induced demand framework suggests that the increased reporting of autism cases may be systematically related to amplified health grants through these institutional and professional incentive structures rather than reflecting genuine epidemiological changes.

This misdiagnosis phenomenon entwines with the burden on the health sector, generating a complex situation as there is an increased prevalence of coexisting physical health conditions in autistic individuals when contrasted with the broader population (87–89). The increased reporting of autism cases could be related to amplified net health grants, establishing a link where heightened funding might inadvertently lead to an uptick in reported autism cases. Notably, data specifies that adults on the autism spectrum, in contrast to their non-autistic counterparts, display a higher probability of receiving diagnoses for conditions such as epilepsy, cardiovascular diseases encompassing dyslipidemia and hypertension, along diabetes (77, 90). Moreover, individuals with ASD face a heightened risk of premature mortality in comparison to the general population. Considering data from two extensive nationwide population-based Swedish registers, documented significantly elevated mortality rates amongst autistic individuals when compared to the general population (91). This intricate connection highlights the importance of careful consideration in the allocation of resources, stressing the need for accurate diagnoses to evade the inflation of autism statistics. This study advances beyond descriptive accounts from prior research by providing both longitudinal analysis and a comprehensive theoretical framework to examine whether well-intentioned healthcare aid policies may produce unintended diagnostic consequences, challenging the widespread assumption that increased mental health funding automatically leads to improved diagnostic accuracy.

### 2.3 Sub-sample analysis of healthcare aids received and autism incidence

To determine the potential existence of overdiagnosis, it is imperative to establish a consistent and distinct definition while also differentiating it from other related issues. Furthermore, the development of effective assessment methods is crucial for gauging the prevalence of overdiagnosis accurately. In light of these considerations, our research endeavors to inspect the correlation between net official development assistance and official aid allocated for healthcare by countries and the reported cases of ASD. This study extends across various dimensions, including the stratification of countries in terms of their mental health services, sub-sample analysis considering income levels, scrutiny of health expenditure, examination of government effectiveness, and exploration of The Economist Democracy Index.

Researchers emphasize the pivotal role of mental health services in influencing the prevalence and reporting patterns of such disorders (92, 93). The stratification based on income levels draws theoretical backing from economic frameworks, aligning with the principles posited by Kawachi et al. (94), which emphasize the substantial influence of financial resources and healthcare accessibility on the identification and reporting of health-related conditions. The investigation of health expenditure finds theoretical grounding in the Health Belief Model, initially proposed by Rosenstock (95) wherein people are predisposed to search for diagnosis and treatment for conditions perceived as severe and necessitating financial investment. The analysis of government effectiveness is theoretically reinforced by Institutional Theory (96), which posits that the efficacy of governance structures significantly affects the implementation of health policies and hence, the reporting patterns of developmental disorders. In addition, the consideration of The Economist Democracy Index presents a political lens, acknowledging the role of democratic governance in nurturing transparency and inclusivity in healthcare reporting (97). This configuration with established theories and empirical evidence highlights the scholarly rigor of our study, aiming to contribute nuanced insights into the intricate relationships between external aid for healthcare and reported autism cases, while accounting for diverse socio-economic and political contexts.

## 3 Methodology

### 3.1 Empirical methodology and baseline model specification

To examine the effect of official aid assistance on ASD, the following baseline line is developed (Equation 1):


(1) $$AUT_{i,t}=\;\beta_{1}(ODA_{it})+\;\beta_{2}(X_{it})+\;\mu_{i}+\;\gamma_{t}+\epsilon_{it}$$

where AUT signifies the total, female, and male autism cases in country i and time t. ODA_it_ means the official development assistance received, and X_it_ encompasses a set of control variables on behalf of various factors such as environmental influences, maternal health, mental health policies, government effectiveness, out-of-pocket expenditures, population size, sex ratio at birth, and gross domestic product. This baseline model incorporates country and time-fixed effects denoted by $\mu_{i}$ and $\gamma_{t}$ , respectively to account for unobserved heterogeneity. The error term is denoted by $\epsilon_{it}$ . The assortment of this model is grounded in theoretical considerations and is aligned with empirical research suggesting potential links between development assistance and health outcomes (98, 99). The utilization of fixed effects and control variables augments the internal validity of the model, allowing for a more nuanced scrutiny of the impact of ODA on autism prevalence, while concurrently accounting for other influential factors. This comprehensive empirical methodology warrants a rigorous analysis of the relationship between development assistance and autism, providing valuable insights into the potential determinants of autism prevalence worldwide.

### 3.2 Endogeneity concern and instrumental variable

Conventional ordinary least squares (OLS) estimation cannot adequately address the endogeneity concerns inherent in examining the relationship between official aid and autism spectrum disorder cases. The endogeneity problem manifests through bidirectional causality, whereby aid may enhance autism reporting via improved diagnostic infrastructure and training, while simultaneously, countries demonstrating higher autism prevalence may attract increased targeted health assistance from donors responding to documented epidemiological needs. Furthermore, omitted variable bias emerges from unobserved heterogeneity in healthcare system quality, government effectiveness, and international engagement levels that concurrently influence both aid allocation decisions and diagnostic capacity for autism spectrum disorders. These confounding factors preclude the isolation of the true causal effect of official aid on reported autism cases, as the relationship operates through multiple interconnected and potentially simultaneous pathways.

To address these methodological challenges, we employ instrumental variable two-stage least squares (IV-2SLS) estimation with fixed effects. Lagged values of official aid serve as instrumental variables based on the theoretical justification that past aid allocations are correlated with current aid levels (satisfying the relevance condition) but are predetermined and thus uncorrelated with contemporary unobserved factors affecting current autism reporting (satisfying the exogeneity condition). The utilization of lagged values as instrumental variables is well-established in the empirical literature, particularly in contexts where identifying truly exogenous external instruments proves methodologically challenging or theoretically implausible (100–102).

The validity and strength of our instrumental variables are rigorously assessed through comprehensive diagnostic testing: first-stage relevance is evaluated using Sanderson-Windmeijer F-statistics and Chi-square tests, overidentification restrictions are tested via Hansen J-statistics to ensure instrument exogeneity, under identification is examined through Kleibergen-Paap rank LM tests to confirm instrument relevance, and weak instrument concerns are addressed using Kleibergen-Paap rank F-statistics to verify sufficient instrument strength for reliable inference.

### 3.3 Data description and summary of statistical techniques

#### 3.3.1 Data description

We have considered country-level autism cases from the Institute for Health Metrics and Evaluation (IHME). Our data set comprised 6180 observations from 206 countries, from the year 1990 to 2019. The details of the variables’ descriptions and sources of data collection are given in **Table 1**. The official development aid received by the country for healthcare initiatives was the dependent variable and the data was collected from the World Bank and measured in constant US$. The reported cases of autism spectrum disorder were considered inclusive of those with autism, Asperger Syndrome, and other autistic spectrum disorders. The cases were measured in total and differentiated by sex. Apart from the main independent variable, several control variables are also incorporated including biological, psychological, and socioeconomic factors to address omitted variable bias. Maternal and neonatal disorders, for instance, play a crucial role in shaping the trajectory of autism cases. Research has constantly shown that prenatal complications, such as maternal infections and nutritional deficiencies, may contribute to an increased risk of ASD in offspring (103, 104). This emphasizes the significance of understanding and addressing maternal health as a key determinant in the prevalence of autism cases. Therefore, in this research maternal and neonatal disorders were assessed in the form of a principal component (PC) derived from three health constructs: communicable, maternal, neonatal, and nutritional diseases; maternal and neonatal disorders; and neonatal disorders specifically. Furthermore, the construction and implementation of mental health policies at the state level significantly impact the reported cases of autism. Nations with comprehensive mental health policies are well-equipped to sense, diagnose, and offer appropriate interventions for individuals with ASD. The absence or inadequacy of such policies may result in misreporting and false identification of autism cases (105). This highlights the need for robust mental health infrastructure and policies to effectively address the challenges posed by autism.

**Table 1:** Description of variables and data sources.

Variables	Definition	Source
Incidence of Autism	The total number of documented instances of autism spectrum disorder reported across all countries during the period spanning from 1990 to 2019.	IHME
Males with Autism	The aggregate number of individuals identified as males diagnosed with autism spectrum disorder within a specified population or sample.	IHME
Females with Autism	The cumulative count of individuals identified as females diagnosed with autism spectrum disorder within a defined population or sample.	IHME
Official Aids Received	The total monetary assistance officially received by a specified entity, typically a country, and explicitly designated for healthcare purposes. This includes funds officially recorded and reported within a specific timeframe to support health-related initiatives.	WDI
Particulate Matter	The concentration of fine particles suspended in the air, often measured as particulate matter (PM), reflects air quality and potential health risks.	WDI
Maternal/Neonatal Disorders	A composite variable was generated by taking a Principal Component of three constructs data, including Communicable, Maternal, Neonatal, and Nutritional diseases; Maternal and Neonatal disorders; and Neonatal disorders.	IHME
Mental Health Policy	A binary metric indicating the presence (1) or absence (0) of a formal mental health policy within a given context, reflecting a governmental approach to mental health issues.	WHO-GHO
Government Effectiveness	Government Effectiveness measures perceptions regarding public service quality, civil service competence and political autonomy, policy development and execution effectiveness, and the credibility of governmental commitment to policy implementation. The indicator is expressed as a standardized score following a normal distribution, with values typically ranging from approximately -2.5 to 2.5 standard deviations.	WGI
Out of Pocket expenditures	The total amount of money spent directly by individuals when seeking healthcare services is not covered by insurance or other third-party payment mechanisms.	WDI
Population Size	The total number of individuals residing in a specific geographic area or defined population group.	WDI
Sex Ratio at Birth	The ratio of male to female births within a given population is typically expressed as the number of male births per 100 female births.	WDI
Gross Domestic Product	The total market value of all goods and services produced within the borders of a country within a specific period is often used as a key indicator of economic health.	WDI

WDI, World development indicators by World bank; IHME, Institute for Health Metrics and Evaluation, Global Burden of Disease; WHO-GHO, World Health Organization-Global Health Indicators.

Government effectiveness, as measured by the sub-component of World Governance Indicators (WGI), is one more critical factor influencing reported autism cases. Efficient and responsive governance guarantees the allocation of resources for early intervention programs, research initiatives, and support services, consequently positively affecting the accuracy and comprehensiveness of autism reporting (106). The theoretical underpinning stands in the role of governance in shaping public health outcomes, emphasizing the necessity for effective governance to alleviate the impact of autism. Socioeconomic factors, such as out-of-pocket expenditures for healthcare, likewise play a role in reported cases of autism. Families with limited financial resources may experience challenges in accessing diagnostic and therapeutic services, possibly leading to misreporting in economically disadvantaged regions (107). Population size, sex ratio at birth, and gross domestic product (GDP) are additional aspects that can impact the reported cases of autism. Larger populations may exhibit higher absolute numbers of autism cases, while variations in sex ratio at birth may contribute to gender-specific differences in prevalence (108–110). Moreover, the GDP of a country correlates with its healthcare infrastructure and resources, influencing the discovery and reporting of autism cases (111).

In conclusion, the reported cases of autism are related to a multitude of factors, encompassing maternal and neonatal health, mental health policies, government effectiveness, socioeconomic variables, and demographic indicators. Understanding and addressing these factors through a multidimensional method is crucial for truthful reporting, effective intervention, and the development of targeted policies to support individuals with ASD and their families. This all-inclusive perspective aligns with the bio-psycho-social model of health (112, 113), highlighting the need to consider biological, psychological, and social determinants to comprehend and address the complexity of autism prevalence.

#### 3.3.2 Summary statistics

**Table 2** presents summary statistics showing substantial variability in autism prevalence with males demonstrating significantly higher rates than females, consistent with existing literature. Official aid varies considerably across entities, while approximately half have formal mental health policies in place. Control variables include government effectiveness, out-of-pocket health expenditures, population sizes, and a principal component of maternal and neonatal disorders derived from communicable, maternal, neonatal, and nutritional disease constructs.

**Table 2:** Summary statistics.

Variables	Obs	Mean	Std. dev.	p25	p75
Prevalence of Autism	6,180	120015.4	431070.9	4399.3	75270.2
Males with Autism	6,180	92175.05	337403	3313.2	56423.7
Females with Autism	6,180	27840.32	96188.78	1069.6	18131.7
Official Aid Received	4,558	4.78E+08	8.88E+08	54100000	5.45E+08
Particulate Matter	6,180	1168.829	818.9256	584.9	1557.8
Maternal/Neonatal Disorders	6,000	-1.34E-10	1.722762	-0.39	-0.155
Mental Health Policy	6180	0.0436893	0.2044195	0.0	1.0
Government Effectiveness	3,475	-2.24E-09	2.238136	-1.6	-1.7
Out of Pocket expenditures	3,716	34.08704	19.69227	17.2	48.6
Population Size	6,120	2375316	9410311	49402	1309950
Sex Ratio at Birth	6,120	105.2536	2.232476	103.8	106.3
Gross Domestic Product	5,640	12770.17	20017.89	1475.4	14664.2

## 4 Empirical results

### 4.1 Preliminary analysis

Benford’s Law and the Mean Absolute Deviation (MAD) are statistical tools that fulfill a central objective, which is the possibility of anomalies inherent in reported global incidences of autism. Benford’s Law inspects the distribution of leading digits in numerical data, and deviations from this law may specify irregularities or anomalies. Likewise, we employed another robust analysis called, MAD, a measure of the dispersion of data points from the mean, providing insights into the overall inconsistency of a dataset. Our implementation pools autism case data across all countries within each income group over the 2015–2019 period, generating sufficient observations (1,920 for low-income and 4,260 for high-income countries) for reliable first-digit distribution analysis rather than conducting yearly country-specific tests. The rationale behind selecting the years 2015 to 2019 for the analysis of global autism cases is grounded in a cautious strategy to examine a post-Diagnostic and Statistical Manual of Mental Disorders, Fifth Edition (DSM-5) era, intentionally circumventing the instantaneous repercussions of the DSM-5’s release in 2013. This step delivers a unique opportunity for a nuanced exploration of inclinations and patterns in reported autism cases, independent of the immediate impact stemming from the revised diagnostic criteria introduced by the DSM-5. The research justification for this timeframe lies in its potential to disclose stabilized and assimilated diagnostic practices on a global scale. Moving past the initial adjustment period to the new criteria, healthcare systems, clinicians, and researchers are expected to have adapted to the DSM-5 guidelines, potentially influencing case identification and reporting practices. By concentrating on the years 2015 to 2019, the analysis aims to encapsulate a duration during which the field has possibly acclimated to the DSM-5 revisions. Benford’s Law, applied to the leading digits of the observed values, is a method to assess the conformity of the data distribution to a theoretically expected pattern. Moreover, MAD assisted as a valuable metric to gauge the average absolute difference of observed values from their mean. This indicates the overall variability in reported autism cases.


$$\;Mean\;Absolute\;Deviation=\;\frac{1}{N}\sum_{1}^{N}fi\lfloor Xi-\bar{X}\rfloor$$

Given the concerning spike in multiple developmental disorders observed during 2016-2017 (**Figure 2**), our analysis focuses on the 2015–2019 period to capture these anomalous patterns while allowing sufficient post-DSM-5 (2) adjustment time. Benford’s Law violations are assessed using chi-square goodness-of-fit tests comparing observed versus expected first-digit distributions across 1,920 low-income and 710 high-income country observations. The chi-square statistic measures the magnitude of deviation from Benford’s theoretical distribution, with higher values indicating greater irregularities. Low-income countries show χ² = 72.91 (*p* = 0.694) compared to high-income countries at χ² = 37.09 (*p* = 0.695), indicating substantially greater systematic deviations from expected patterns. Similarly, MAD analysis reveals low-income countries exhibit nearly double the variability (1.647 *vs* 0.830), corroborating systematic differences in reporting patterns. These statistical disparities suggest potential data quality concerns in autism prevalence reporting, particularly within resource-constrained settings where external incentives may influence diagnostic practices.

**Figure 3:**
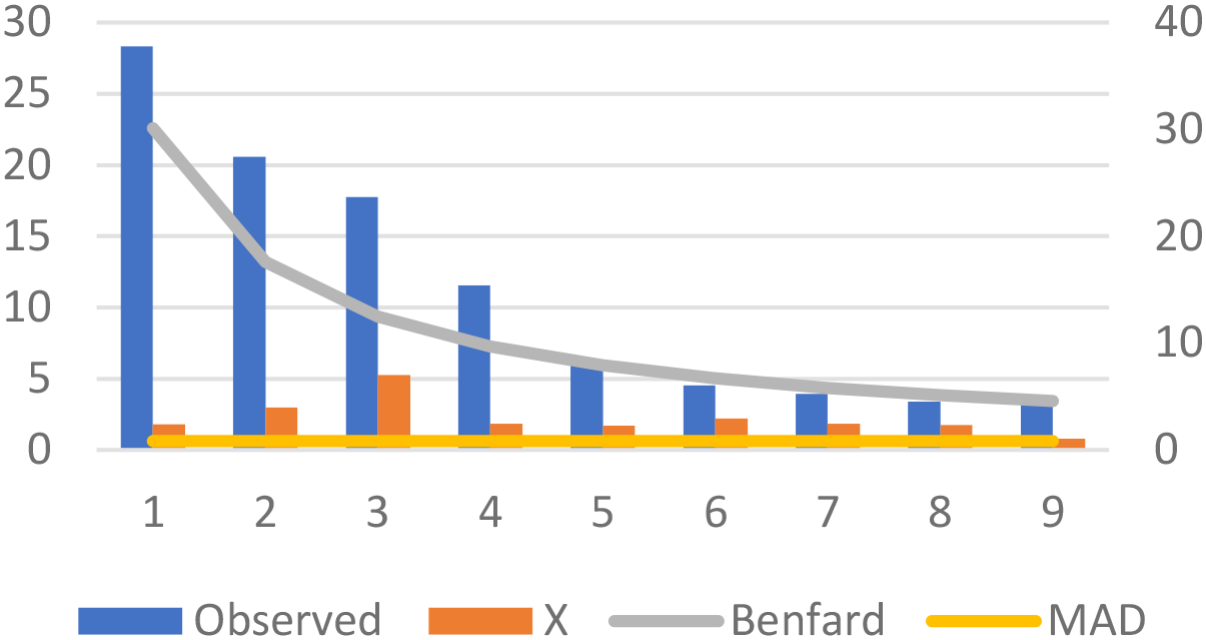
Autism prevalence in high-income countries (2015-2019): MAD and Benford’s Law compliance.

**Figure 4:**
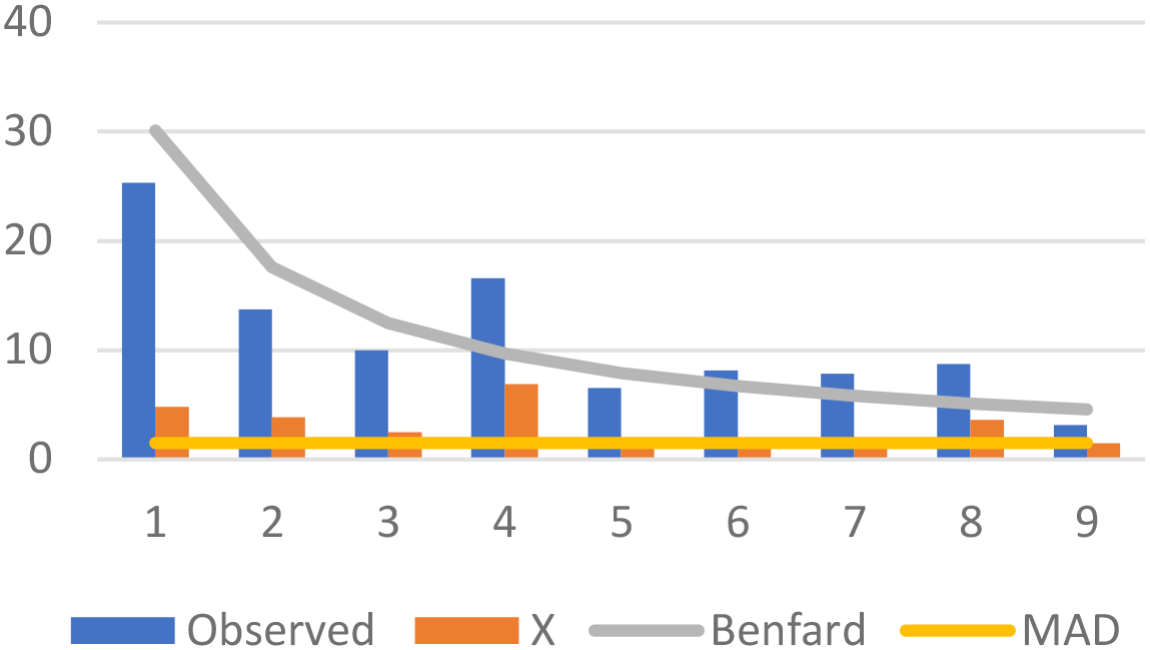
Autism prevalence in low-income countries (2015-2019): MAD and Benford’s Law compliance.

The low-income countries’ dataset discloses varying levels of deviation from the mean, as reflected by the calculated MAD values. Meanwhile, Benford’s Law analysis on the leading digits allows for an additional layer of scrutiny, recognizing potential irregularities in the distribution of observed values. These analytical techniques assist as valuable tools for identifying patterns, trends, or discrepancies that warrant further investigation. Likewise, high-income countries undergo the same analytical process. MAD values provide insights into the dispersion of reported autism cases from the mean, while Benford’s Law analysis sheds light on the distribution of leading digits. A comparative analysis amongst the low and high-income countries’ datasets can further clarify potential disparities or similarities in the reported autism cases. Upon graphically representing these datasets, the visualizations display the trends, deviations, and distributions (**Figures 3**, **4**).

### 4.2 Main results

**Table 3** elaborates on the baseline proposition behind this research which is the influence of official aid received on the prevalence of autism spectrum disorder. The study discovers this relationship by employing two models: Model 1, which eliminates fixed effects, and Model 2, which includes both year and country fixed effects. In Model 1, the focal variable, Official Aid Received, appears as a statistically significant predictor of the prevalence of autism across all categories, overall prevalence, male and female prevalence. The coefficients of 0.085, 0.084, and 0.088 for overall prevalence, male, and female prevalence, respectively, indicate a high statistical significance. This infers that, on average, a one-unit increase in official aid received is associated with the corresponding increase in prevalence across the specified categories. Moving to Model 2, which accounts for fixed effects, the robustness of the relationship between official aid received and ASD prevalence is constant. The coefficients rise slightly to 0.094, 0.094, and 0.096 for overall prevalence, male prevalence, and female prevalence, respectively, and retain their high level of significance. The inclusion of fixed effects increases the model’s control for potential confounding factors, reinforcing the integrity of the findings.

**Table 3:** The impact of official aid received on the prevalence of autism spectrum disorder.

	Model 1 (without fixed effect)			Model 2 (with fixed effect)		
Variables	Autism	Males	Females	Autism	Males	Females
Official Aid Received	0.085***	0.084***	0.088***	0.094***	0.094***	0.096***
	(0.020)	(0.021)	(0.019)	(0.020)	(0.021)	(0.020)
Particulate Matter	0.030*	0.022	0.053***	0.022	0.013	0.047***
	(0.018)	(0.018)	(0.017)	(0.018)	(0.019)	(0.017)
Maternal/Neonatal Disorders	0.049***	0.048***	0.052***	0.049***	0.048***	0.052***
	(0.004)	(0.005)	(0.004)	(0.004)	(0.005)	(0.004)
Mental Health Policy	-0.008	-0.008	-0.007	0.023	0.026	0.016
	(0.029)	(0.030)	(0.028)	(0.030)	(0.031)	(0.029)
Government Quality	-0.095***	-0.097***	-0.091***	-0.100***	-0.102***	-0.094***
	(0.007)	(0.007)	(0.007)	(0.007)	(0.007)	(0.007)
Out of Pocket expenditures	-0.020	-0.028	0.010	-0.031	-0.039	0.001
	(0.038)	(0.038)	(0.036)	(0.038)	(0.039)	(0.036)
Population Size	0.872***	0.871***	0.874***	0.872***	0.872***	0.874***
	(0.005)	(0.006)	(0.005)	(0.005)	(0.006)	(0.005)
Sex Ratio at Birth	-0.653***	-0.618***	-0.761***	-0.650***	-0.614***	-0.759***
	(0.028)	(0.029)	(0.027)	(0.028)	(0.029)	(0.027)
Gross Domestic Product	-0.125***	-0.114***	-0.160***	-0.121***	-0.109***	-0.157***
	(0.009)	(0.010)	(0.009)	(0.009)	(0.010)	(0.009)
Year Fixed Effect				YES	YES	YES
Country Fixed Effect				YES	YES	YES
Constant	-0.653	-1.001**	-1.935***	10.271***	10.756***	6.278**
	(0.473)	(0.482)	(0.451)	(2.864)	(2.920)	(2.742)
Observations	2,239	2,239	2,239	2,239	2,239	2,239

Government Quality (GQ PCA of 5 factors namely, Violence and accountability, Political Stability and Absence of Violence/Terrorism, Government Effectiveness, Regulatory Quality, Corruption from World bank Data Source. Standard errors in parentheses, ***p<0.01, **p<0.05, *p<0.1.

The positive relationship between official aid received and reported autism incidence identified in this study reflects broader patterns documented in the literature regarding unintended consequences of targeted healthcare funding. This finding aligns with research by Sarr et al. (84), who emphasize how increased funding to specific healthcare sectors can produce inadvertent effects on diagnostic practices. Similarly, the economic impact literature demonstrates how resource influxes can alter healthcare delivery patterns, as shown by Smith et al. (114) in their analysis of pandemic influenza funding effects on health outcomes. Marty et al. (115), further illuminate these dynamics through their examination of healthcare resource allocation, particularly highlighting how financial resource surges can lead to unintended consequences including over-diagnosis tendencies.

**Figure 5:**
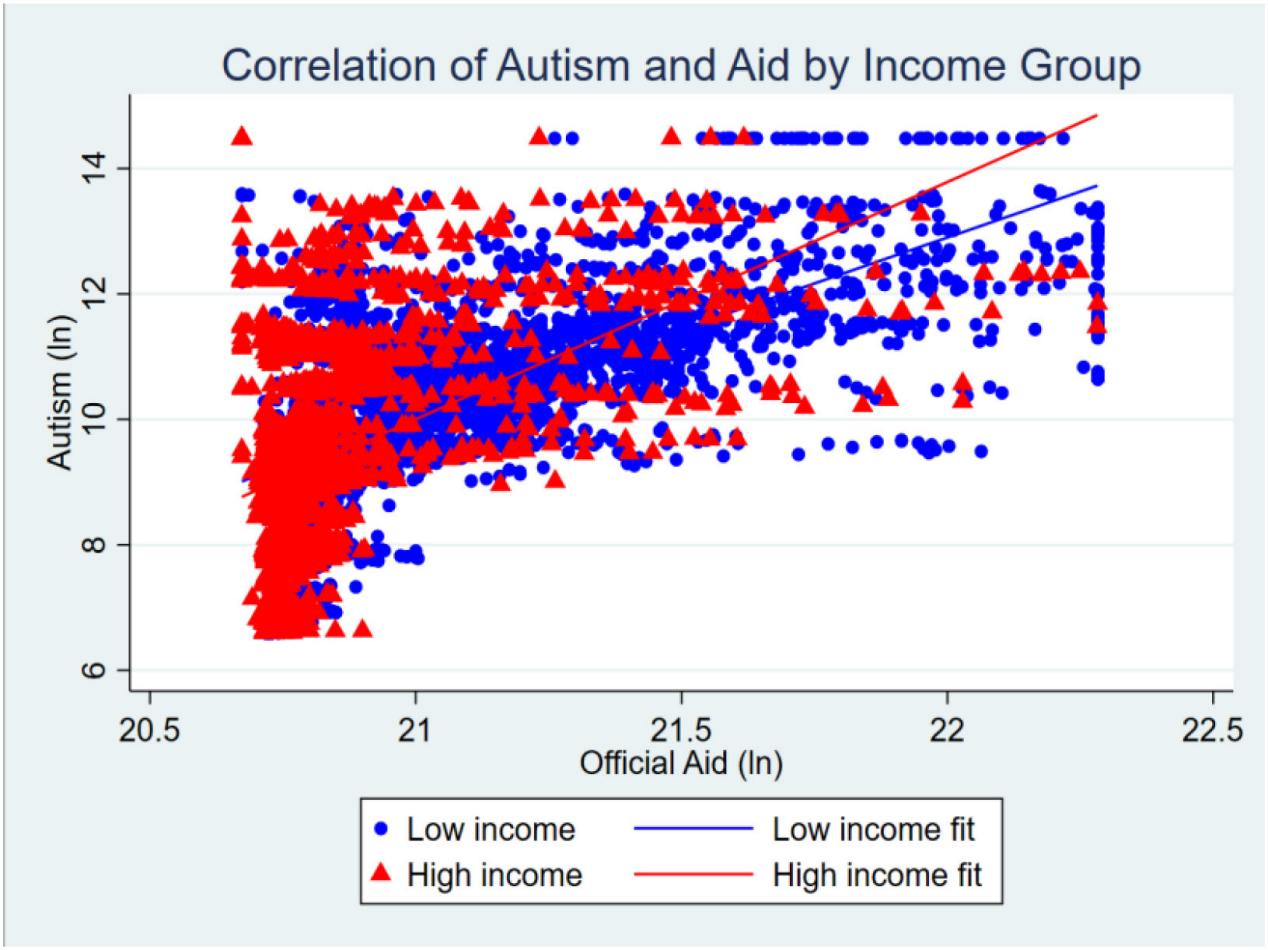
Scatter plot of correlation between official aid and autism prevalence by income group.

The scatter plot analysis (**Figure 5**) visually demonstrates the differential correlation patterns observed in our statistical models. Low-income countries show a positive correlation between official aid and autism prevalence, while high-income countries display a less pronounced association. These observed patterns are consistent with our regression findings across income groups, though the correlation analysis alone cannot distinguish between genuine diagnostic improvement and potential reporting irregularities. The visual data supports the statistical evidence of systematic differences between income groups but requires interpretation alongside our broader analytical framework that includes controls for institutional capacity and healthcare infrastructure to understand the underlying mechanisms driving these relationships. Given these differential patterns, we conducted comprehensive sub-sample analyses to examine how various country characteristics moderate the aid-autism relationship, the results of which we report in the following sections.

**Table 4** presents sub-sample analysis results examining how mental health service availability moderates the relationship between official aid and autism prevalence. The analysis divides countries into low and high mental health services groups based on median values (≤8.03 *vs >*8.03). In low mental health service countries, official aid demonstrates statistically significant positive associations with overall autism prevalence, female autism prevalence, and male autism prevalence. Conversely, in high mental health service countries, these associations become non-significant, indicating that robust mental health infrastructure attenuates the aid-prevalence relationship. Control variables generally behave as expected, with government effectiveness showing negative associations and population size showing positive associations across both groups.

**Table 4:** The impact of official aid received on the prevalence of autism spectrum disorder: sub-sample analysis of countries with high and low mental health services.

	Low mental health services			High mental health services		
Variables	Autism	Females	Males	Autism	Females	Males
Official Aid Received	0.125***	0.132***	0.125***	-0.150***	-0.174***	-0.142***
	(0.021)	(0.020)	(0.022)	(0.043)	(0.041)	(0.043)
Particulate Matter	0.002	0.010	-0.002	0.036	0.072***	0.024
	(0.025)	(0.023)	(0.025)	(0.026)	(0.025)	(0.027)
Maternal/Neonatal Disorders	0.027***	0.031***	0.025***	0.127***	0.135***	0.124***
	(0.005)	(0.004)	(0.005)	(0.011)	(0.011)	(0.011)
Mental Health Policy	0.029	0.021	0.032	0.015	0.008	0.017
	(0.036)	(0.033)	(0.037)	(0.045)	(0.043)	(0.046)
Government Quality	-0.084***	-0.077***	-0.086***	-0.121***	-0.117***	-0.123***
	(0.009)	(0.009)	(0.010)	(0.010)	(0.010)	(0.010)
Out of Pocket expenditures	-0.248***	-0.230***	-0.250***	0.072	0.125**	0.056
	(0.049)	(0.046)	(0.050)	(0.056)	(0.054)	(0.057)
Population Size	0.892***	0.897***	0.890***	0.848***	0.848***	0.848***
	(0.007)	(0.006)	(0.007)	(0.008)	(0.008)	(0.009)
Sex Ratio at Birth	-0.513***	-0.666***	-0.464***	-0.714***	-0.792***	-0.688***
	(0.037)	(0.034)	(0.038)	(0.042)	(0.040)	(0.042)
Gross Domestic Product	-0.120***	-0.155***	-0.108***	-0.046***	-0.082***	-0.035**
	(0.012)	(0.011)	(0.012)	(0.016)	(0.015)	(0.016)
Year Fixed Effect	YES	YES	YES	YES	YES	YES
Country Fixed Effect	YES	YES	YES	YES	YES	YES
Constant	16.034***	12.824***	16.266***	12.048***	7.360*	12.705***
	(3.550)	(3.311)	(3.646)	(4.158)	(3.995)	(4.240)
Observations	1,181	1,181	1,181	1,058	1,058	1,058

Mental Health Services are calculated by adding three aspects (Psychiatrists working in the mental health sector, Nurses working in the mental health sector (per 100–000 population), Social workers working in the mental health sector, Psychologists working in the mental health sector (per 100–000 population) The group was divided into categories based on Median=8.03. Standard errors in parentheses ***p<0.01, **p<0.05, *p<0.1.

These findings directly support the supplier-induced demand theory outlined in our theoretical framework, where healthcare funding creates systematic incentives that influence diagnostic patterns through infrastructure development, professional training, and service expansion (79, 80). This aligns with research by Greenspoon and Saklofske (116) Matson and Kozlowski (117), and Zablotsky et al. (118), who highlighted the critical role that mental health service availability and convenience play in ensuring correct autism diagnoses. The significant aid-prevalence relationship in low-service countries exemplifies how funding-dependent training programs may emphasize autism recognition over differential diagnosis, creating confirmation bias toward autism symptoms rather than alternative explanations (84). This is particularly concerning given documented overlapping behavioral features between ASD, Down Syndrome, and ADHD that complicate clinical assessments (60, 62). The moderation effect validates our theoretical proposition that autism-specific services funded through targeted aid create systematic incentives for autism diagnoses over other developmental conditions lacking equivalent funding streams.

In well-resourced healthcare systems with robust mental health infrastructure, standardized diagnostic protocols and professional training reduce susceptibility to these funding influences, explaining the non-significant results in high-service countries. Conversely, in resource-constrained settings, the three-channel mechanism operates more prominently, where increased funding enhances diagnostic capacity without corresponding improvements in differential diagnostic rigor. These results underscore the importance of context-specific approaches to healthcare aid allocation, where policymakers should prioritize establishing robust diagnostic frameworks and professional training standards before expanding screening initiatives in resource-poor settings.

**Table 5** explains the relationship between official aid and autism prevalence across four income-level country categories. The results reveal contrasting patterns across income groups. In high-income countries, official aid demonstrates significant negative associations with autism prevalence (β = -0.519 for total, -0.584 for females, -0.508 for males; all p < 0.001). Upper-middle-income countries show similar negative effects with reduced magnitude. The relationship reverses in lower-middle-income countries, where positive associations emerge (β = 0.370, 0.378, 0.367 respectively; all p < 0.001), with lower-income countries displaying comparable positive patterns with smaller coefficients.

**Table 5:** The impact of official aid received on the prevalence of ASD: sub-sample analysis for the level of income.

	High income			Upper middle income			Lower middle income			Lower income		
Variables	Autism	Females	Males	Autism	Females	Males	Autism	Females	Males	Autism	Females	Males
Official Aid	-0.519***	-0.584***	-0.508***	-0.064**	-0.053**	-0.067**	0.370***	0.378***	0.367***	0.167***	0.152***	0.173***
	(0.141)	(0.122)	(0.148)	(0.026)	(0.026)	(0.026)	(0.046)	(0.047)	(0.046)	(0.032)	(0.032)	(0.031)
Particulate Matter	-0.060	-0.017	-0.073	0.009	0.036	-0.000	-0.077	-0.024	-0.094**	0.022	0.038	0.016
	(0.057)	(0.050)	(0.060)	(0.025)	(0.026)	(0.026)	(0.047)	(0.047)	(0.047)	(0.031)	(0.032)	(0.031)
Maternal/NeonatalDisorders	0.537	1.483**	0.218	0.427***	0.367***	0.445***	0.031***	0.036***	0.029***	0.255***	0.269***	0.251***
	(0.660)	(0.572)	(0.695)	(0.024)	(0.024)	(0.024)	(0.005)	(0.005)	(0.005)	(0.028)	(0.029)	(0.028)
Mental Health Policy	0.017	0.010	0.018	0.029	0.019	0.033	0.021	0.016	0.022	0.041	0.047	0.039
	(0.114)	(0.099)	(0.120)	(0.038)	(0.039)	(0.039)	(0.046)	(0.047)	(0.047)	(0.039)	(0.040)	(0.039)
Government Quality	-0.261***	-0.217***	-0.274***	-0.075***	-0.074***	-0.075***	-0.130***	-0.115***	-0.135***	-0.022**	-0.004	-0.028***
	(0.027)	(0.024)	(0.029)	(0.010)	(0.010)	(0.010)	(0.013)	(0.013)	(0.013)	(0.011)	(0.011)	(0.011)
Out of Pocket Exp	0.598***	0.546***	0.612***	-0.024	-0.017	-0.024	-0.316***	-0.197***	-0.354***	0.020	0.039	0.013
	(0.124)	(0.108)	(0.131)	(0.053)	(0.054)	(0.054)	(0.072)	(0.073)	(0.072)	(0.059)	(0.060)	(0.059)
Population Size	0.931***	0.899***	0.943***	0.823***	0.835***	0.819***	0.865***	0.858***	0.868***	0.797***	0.791***	0.799***
	(0.022)	(0.019)	(0.023)	(0.008)	(0.008)	(0.008)	(0.015)	(0.016)	(0.015)	(0.016)	(0.016)	(0.016)
Sex Ratio at Birth	-1.147***	-0.944***	-1.208***	-0.597***	-0.740***	-0.551***	-0.721***	-0.845***	-0.681***	-0.215***	-0.295***	-0.187***
	(0.203)	(0.176)	(0.214)	(0.037)	(0.038)	(0.038)	(0.044)	(0.044)	(0.044)	(0.050)	(0.051)	(0.050)
Gross Domestic Product	0.721***	0.504***	0.782***	-0.059***	-0.095***	-0.047**	-0.060**	-0.078***	-0.054**	-0.096***	-0.120***	-0.088***
	(0.056)	(0.048)	(0.059)	(0.020)	(0.021)	(0.021)	(0.024)	(0.024)	(0.024)	(0.018)	(0.019)	(0.018)
Year Fixed Effect	YES	YES	YES	YES	YES	YES	YES	YES	YES	YES	YES	YES
Country Fixed Effect	YES	YES	YES	YES	YES	YES	YES	YES	YES	YES	YES	YES
Constant	55.039***	49.245***	55.911***	16.501***	14.574***	16.411***	9.008*	6.674	9.104*	-11.281***	-14.327***	-10.997***
	(11.685)	(10.121)	(12.308)	(4.018)	(4.053)	(4.046)	(4.886)	(4.959)	(4.891)	(3.833)	(3.938)	(3.820)
Observations	230	230	230	970	970	970	424	424	424	615	615	615

The countries were divided into High Income, Upper Middle Income, Lower Middle Income, Lower Income Countries based on statistics of World bank statistics. Standard errors in parentheses ***p<0.01, **p<0.05, *p<0.1.

This income-stratified pattern provides compelling evidence for the Economic Theory of Health Behavior and aligns with research by Kawachi et al. (94), highlighting how financial resources influence healthcare identification and reporting. The findings support broader literature on diagnostic trends (119–121), identifying systematic overuse of healthcare facilities particularly prevalent in low- and middle-income countries. This pattern reflects the three-channel mechanism we established theoretically, where funding influxes in resource-constrained settings create diagnostic capacity without corresponding improvements in differential diagnostic frameworks. As Born et al. (122) and Albarqouni et al. (123) demonstrate, augmented attention to health issues through external funding can intensify diagnostic efforts, potentially leading to the positive aid-prevalence associations observed in lower-income settings. The WHO’s call for context-specific strategies to address overdiagnosis (124) becomes particularly relevant here, suggesting that aid effectiveness depends critically on existing healthcare infrastructure and institutional capacity rather than funding levels alone.

**Table 6** is revealing distinct patterns that support our theoretical framework on healthcare resource allocation. In low health expenditure countries, official aid demonstrates significant positive associations with autism prevalence (*β* = 0.239 for total, 0.231 for females, 0.242 for males; all *p <* 0.001). This unexpected positive association suggests a complex relationship between official aid and reported ASD prevalence in countries with limited healthcare spending. Medium health expenditure countries show no significant associations, with coefficients near zero and small standard errors, demonstrating a lack of meaningful connection between official aid and reported ASD prevalence. High health expenditure countries display significant negative associations (*β* = -0.032 for total, -0.064 for females, -0.026 for males; *p* < 0.05), creating a clear gradient from positive to negative effects as health expenditure capacity increases. This negative association aligns more closely with expectations, suggesting that in countries with high health expenditure, financial assistance may contribute to lower reported ASD prevalence.

**Table 6:** The impact of official aid received on the prevalence of autism spectrum disorder: sub-sample analysis for the level of health expenditure.

	Low health expenditure			Medium health expenditure			High health expenditure		
Variables	Autism	Females	Males	Autism	Females	Males	Autism	Females	Males
Official Aids Received	0.239***	0.231***	0.242***	-0.000	0.015	-0.004	-0.032	-0.064	-0.026
	(0.030)	(0.030)	(0.030)	(0.025)	(0.024)	(0.026)	(0.130)	(0.100)	(0.141)
Particulate Matter	-0.230***	-0.184***	-0.246***	0.086***	0.092***	0.083***	0.244**	0.263***	0.234**
	(0.028)	(0.029)	(0.029)	(0.023)	(0.022)	(0.023)	(0.108)	(0.083)	(0.117)
Maternal/NeonatalDisorders	0.028***	0.033***	0.027***	0.222***	0.210***	0.224***	0.243*	0.381***	0.202
	(0.004)	(0.004)	(0.004)	(0.014)	(0.014)	(0.015)	(0.127)	(0.098)	(0.138)
Mental Health Policy	0.016	0.018	0.016	0.031	0.023	0.034	0.020	0.013	0.023
	(0.040)	(0.040)	(0.040)	(0.037)	(0.035)	(0.038)	(0.072)	(0.055)	(0.078)
Government Quality	-0.047***	-0.030***	-0.053***	-0.107***	-0.106***	-0.108***	-0.238***	-0.185***	-0.253***
	(0.010)	(0.010)	(0.010)	(0.008)	(0.008)	(0.009)	(0.031)	(0.024)	(0.034)
Out of Pocket expenditures	0.210***	0.272***	0.192***	-0.149***	-0.128***	-0.154***	1.560***	1.446***	1.589***
	(0.051)	(0.051)	(0.051)	(0.047)	(0.046)	(0.048)	(0.282)	(0.217)	(0.307)
Population Size	0.826***	0.825***	0.826***	0.857***	0.862***	0.856***	0.953***	0.925***	0.963***
	(0.011)	(0.011)	(0.011)	(0.006)	(0.006)	(0.007)	(0.022)	(0.017)	(0.024)
Sex Ratio at Birth	-0.331***	-0.445***	-0.293***	-0.661***	-0.782***	-0.622***	-1.410***	-1.166***	-1.471***
	(0.036)	(0.036)	(0.036)	(0.035)	(0.034)	(0.036)	(0.250)	(0.192)	(0.271)
Gross Domestic Product	-0.036**	-0.075***	-0.022	-0.067***	-0.105***	-0.055***	0.601***	0.436***	0.645***
	(0.017)	(0.017)	(0.017)	(0.015)	(0.015)	(0.016)	(0.137)	(0.105)	(0.149)
Year Fixed Effect	YES	YES	YES	YES	YES	YES	YES	YES	YES
Country Fixed Effect	YES	YES	YES	YES	YES	YES	YES	YES	YES
Constant	-11.940***	-15.838***	-11.342***	14.022***	11.528***	14.023***	-14.239	-34.953**	-8.528
	(3.383)	(3.400)	(3.404)	(3.837)	(3.689)	(3.914)	(18.978)	(14.586)	(20.625)
Observations	792	792	792	1,362	1,362	1,362	85	85	85

The countries were divided into 3 categories based on statistics of Current health expenditure per capita (current US$) World bank statistics. Percentiles were considered for segregation, LHE<25%, MHE>=25% and <=75%, HHE>75% percentile. Standard errors in parentheses ***p<0.01, **p<0.05, *p<0.1.

These findings align with the Health Belief Model framework (95), where healthcare expenditure capacity influences how financial resources translate into diagnostic behavior. In low health expenditure countries, the influx of financial aid likely contributes to heightened global attention on healthcare issues, including neurodevelopmental disorders like autism. Increased awareness and resources might lead to enhanced identification and reporting of ASD cases, possibly resulting in an apparent rise in prevalence, consistent with the overdiagnosis phenomenon (120).

This further aligns with previous research (118, 125) highlighting how awareness and diagnostic practices influence reported ASD prevalence. In medium-expenditure countries, the absence of significant effects suggests these nations maintain a balance in their healthcare systems, with adequate resources for ASD identification and reporting, consequently reducing susceptibility to overdiagnosis due to external financial aid. This interpretation is supported by research from Ehsan et al. (126) and Zeidan et al. (127) underscoring healthcare infrastructure’s importance in influencing ASD prevalence rates. The negative associations in high-expenditure settings raise intriguing questions, suggesting that well-established healthcare systems and diagnostic practices may lead to more accurate identification and reporting of ASD cases, minimizing potential for overdiagnosis. This interpretation aligns with Tekola et al. (19) and Xu et al. (128) emphasizing advanced healthcare infrastructure’s role in refining ASD prevalence estimate accuracy. The unexpected positive association between official aid and reported ASD prevalence in countries with low health expenditure raises important questions about the complex relationship between financial assistance and public health outcomes.

Government effectiveness emerges as a critical moderator in the relationship between official aid and autism prevalence, as demonstrated in **Table 7**’s sub-sample analysis across three governance categories. Countries with low government effectiveness show significant positive associations between official aid and autism prevalence, indicating that higher aid levels correlate with increased reported ASD cases. Medium government effectiveness countries display similar patterns with comparable coefficients, suggesting persistent positive aid-prevalence relationships in moderate governance contexts. However, high government effectiveness countries exhibit dramatically different results, with strong negative associations, where official aid correlates with lower reported autism prevalence.

**Table 7:** The impact of official aid received on prevalence of autism spectrum disorder: sub-sample analysis for level of government effectiveness.

	Low government effectiveness			Medium government effectiveness			High government effectiveness		
Variables	Autism	Females	Males	Autism	Females	Males	Autism	Females	Males
Official Aid Received	0.164***	0.120***	0.180***	0.163***	0.119***	0.178***	-0.990***	-1.022***	-0.988***
	(0.028)	(0.027)	(0.028)	(0.027)	(0.027)	(0.028)	(0.230)	(0.200)	(0.240)
Particulate Matter	-0.005	0.026	-0.016	-0.004	0.027	-0.015	0.206*	0.189**	0.208*
	(0.025)	(0.025)	(0.025)	(0.025)	(0.025)	(0.025)	(0.109)	(0.095)	(0.114)
Maternal/Neonatal Disorders	0.102***	0.105***	0.100***	0.105***	0.109***	0.104***	0.936***	1.079***	0.888***
	(0.010)	(0.010)	(0.010)	(0.010)	(0.010)	(0.010)	(0.238)	(0.208)	(0.249)
Mental Health Policy	0.028	0.028	0.029	0.036	0.034	0.036	-0.039	-0.037	-0.039
	(0.038)	(0.038)	(0.038)	(0.039)	(0.038)	(0.039)	(0.174)	(0.152)	(0.182)
Government Quality	-0.112***	-0.096***	-0.119***	-0.114***	-0.098***	-0.120***	-0.348***	-0.293***	-0.363***
	(0.012)	(0.012)	(0.013)	(0.012)	(0.012)	(0.013)	(0.057)	(0.049)	(0.059)
Out of Pocket expenditures	0.081*	0.104**	0.074	0.079*	0.104**	0.073	0.238	0.200	0.252
	(0.047)	(0.047)	(0.048)	(0.047)	(0.047)	(0.047)	(0.179)	(0.156)	(0.188)
Population Size	0.814***	0.830***	0.809***	0.814***	0.830***	0.809***	0.940***	0.917***	0.948***
	(0.010)	(0.010)	(0.010)	(0.010)	(0.010)	(0.010)	(0.025)	(0.022)	(0.026)
Sex Ratio at Birth	-0.453***	-0.567***	-0.415***	-0.461***	-0.576***	-0.422***	-0.471**	-0.419**	-0.486**
	(0.036)	(0.036)	(0.036)	(0.036)	(0.036)	(0.037)	(0.215)	(0.187)	(0.225)
Gross Domestic Product	-0.123***	-0.147***	-0.114***	-0.122***	-0.147***	-0.114***	0.370***	0.209***	0.413***
	(0.011)	(0.011)	(0.011)	(0.011)	(0.011)	(0.011)	(0.068)	(0.060)	(0.072)
Year Fixed Effect	YES	YES	YES	YES	YES	YES	YES	YES	YES
Country Fixed Effect	YES	YES	YES	YES	YES	YES	YES	YES	YES
Constant	-5.843*	-7.957**	-5.868	-6.216*	-8.314**	-6.247*	52.024***	50.725***	51.589***
	(3.546)	(3.520)	(3.587)	(3.545)	(3.517)	(3.587)	(14.813)	(12.912)	(15.495)
Observations	775	775	775	766	766	766	135	135	135

The countries were divided into 3 categories (High, Medium and Low) based on statistics of Government Effectiveness from World bank Data Source. Percentiles were considered for segregation, Low <25%, Medium >=25% and <=75%, High >75% percentile. Standard errors in parentheses ***p<0.01, **p<0.05, *p<0.1.

This governance-based gradient aligns with Institutional Theory (96) established in our literature, which posits that governance structure efficacy significantly affects health policy implementation and reporting patterns. The positive associations in low and medium effectiveness settings correspond with healthcare system constraints that highlight systematic limitations in accurate symptom detection and reporting capabilities within these governance contexts (74, 119, 124). In these contexts, external aid may serve as critical intervention addressing diagnostic capacity gaps, potentially leading to heightened awareness and increased identification efforts that could contribute to apparent prevalence increases. This mechanism exemplifies our three-channel theoretical framework, where funding influxes create diagnostic infrastructure without corresponding improvements in differential diagnostic rigor, particularly problematic when governance structures cannot effectively oversee diagnostic quality standards. Conversely, the strong negative associations in high effectiveness countries suggest that robust governance structures enable more stringent diagnostic criteria and better control over potential overdiagnosis (35). These countries likely possess the institutional capacity to channel aid toward improving diagnostic accuracy rather than simply expanding identification efforts, reflecting the Social Determinants of Health framework (129), where governance quality shapes how economic resources translate into health outcomes. The governance moderation effect provides compelling evidence that aid effectiveness depends critically on existing institutional capacity to maintain diagnostic standards and quality control mechanisms.

The sub-sample analysis examining official aid’s impact on ASD prevalence across different regime types reveals striking governance-dependent patterns (**Table 8**). Full democracies show significant negative associations while flawed democracies, hybrid regimes, and authoritarian regimes exhibit positive associations. This divergent pattern aligns with institutional theory and the “selectivity hypothesis” that aid works better in environments with strong governance institutions (131–133), Recent evidence also supports this, showing that “the aggregate effect of aid on democracy has become more positive after the Cold War” and that “stable inflows of ‘governance aid’ drive aggregate net positive effects” (134–136). Democratic contexts benefit from “institutional efficiency” (137), where transparency and civil society oversight ensure effective resource allocation toward genuine healthcare improvements (138). This strengthens diagnostic infrastructure and reduces stigma (139, 140). Conversely, non-democratic settings suffer from “extractive institutions” (141) that enable elite capture and strategic manipulation of health statistics. Weaker institutional frameworks result in aid misallocation, limited transparency, and perverse incentives that distort diagnostic practices (132, 142, 143). The absence of democratic accountability creates information asymmetries that facilitate diagnostic inflation and resource misappropriation (144).

**Table 8:** The impact of official aid received on the prevalence of autism spectrum disorder: sub-sample analysis for the level of types of regimes by the economist democracy index.

	Full democracies			Flawed democracies			Hybrid regimes			Authoritarian regimes		
Variables	Autism	Males	Females	Autism	Males	Females	Autism	Males	Females	Autism	Males	Females
Official Aid Received	-0.406**	-0.410***	-0.410**	0.242***	0.232***	0.245***	0.024	0.057	0.013	0.042**	0.044**	0.043**
	(0.167)	(0.154)	(0.174)	(0.046)	(0.047)	(0.046)	(0.045)	(0.044)	(0.045)	(0.021)	(0.019)	(0.021)
Particulate Matter	-0.135**	-0.106*	-0.145**	-0.156***	-0.127***	-0.165***	-0.261***	-0.239***	-0.268***	0.280***	0.260***	0.285***
	(0.066)	(0.061)	(0.069)	(0.028)	(0.028)	(0.028)	(0.049)	(0.048)	(0.049)	(0.033)	(0.031)	(0.034)
Maternal/Neonatal Disorders	0.996***	1.068***	0.970***	0.037***	0.042***	0.036***	0.133***	0.128***	0.134***	0.391***	0.334***	0.407***
	(0.112)	(0.103)	(0.117)	(0.005)	(0.005)	(0.005)	(0.011)	(0.011)	(0.012)	(0.022)	(0.020)	(0.022)
Mental Health Policy	0.024	0.025	0.023	-0.009	-0.008	-0.010	0.040	0.030	0.044	0.060	0.049	0.063
	(0.072)	(0.067)	(0.075)	(0.038)	(0.039)	(0.038)	(0.055)	(0.054)	(0.056)	(0.047)	(0.044)	(0.048)
Government Quality	0.123***	0.105***	0.127***	-0.022	-0.011	-0.026*	0.010	0.011	0.009	-0.008	-0.028**	-0.002
	(0.040)	(0.037)	(0.041)	(0.015)	(0.015)	(0.014)	(0.018)	(0.018)	(0.018)	(0.013)	(0.012)	(0.013)
Out of Pocket expenditure	0.082	-0.068	0.129	0.024	0.136**	-0.012	0.191**	0.247***	0.173*	0.049	0.021	0.058
	(0.101)	(0.093)	(0.105)	(0.053)	(0.054)	(0.053)	(0.088)	(0.087)	(0.089)	(0.059)	(0.056)	(0.061)
Population Size	0.878***	0.870***	0.882***	0.845***	0.847***	0.845***	0.871***	0.873***	0.871***	0.748***	0.759***	0.746***
	(0.017)	(0.016)	(0.018)	(0.009)	(0.010)	(0.009)	(0.011)	(0.010)	(0.011)	(0.010)	(0.010)	(0.011)
Sex Ratio at Birth	0.117	-0.062	0.168	-0.658***	-0.804***	-0.611***	-1.062***	-1.064***	-1.062***	-0.386***	-0.470***	-0.359***
	(0.125)	(0.115)	(0.130)	(0.039)	(0.040)	(0.039)	(0.073)	(0.072)	(0.074)	(0.043)	(0.041)	(0.045)
GDP	-0.425***	-0.432***	-0.423***	-0.117***	-0.146***	-0.106***	-0.142***	-0.205***	-0.121***	-0.153***	-0.180***	-0.145***
	(0.053)	(0.048)	(0.055)	(0.014)	(0.015)	(0.014)	(0.020)	(0.020)	(0.020)	(0.015)	(0.014)	(0.015)
Year Fixed Effect	YES	YES	YES	YES	YES	YES	YES	YES	YES	YES	YES	YES
Country Fixed effect	YES	YES	YES	YES	YES	YES	YES	YES	YES	YES	YES	YES
Constant	YES	YES	YES	YES	YES	YES	YES	YES	YES	YES	YES	YES
Observations	220	220	220	780	780	780	543	543	543	696	696	696

The index used to categorize the countries in groups was taken by Unit (130). Standard errors in parentheses ***p<0.01, **p<0.05, *p<0.1.

The instrumental variable two-stage least squares results (**Table 9**) provide crucial validation of the causal relationship between official aid and autism prevalence, effectively addressing endogeneity concerns that could bias conventional regression estimates. The IV-2SLS approach yields consistently positive and statistically significant coefficients across all specifications, establishing that the observed aid-autism relationship is genuinely causal rather than merely correlational (145). This causal identification definitively rules out reverse causality, where donors might systematically target countries with higher disease burdens, and eliminates concerns about omitted variable bias that could confound the relationship (146). The robust first-stage diagnostics, including F-statistics well above conventional thresholds, confirm instrument strength and validate the reliability of these causal inferences, providing compelling econometric evidence that previous findings reflect true causal effects rather than spurious associations (147).

**Table 9:** Instrumental variable two-stage least square (IV-2SLS) regression estimations.

Variables	Autism	Females	Males
Official Aid Received	0.049***	0.055***	0.047***
	(0.013)	(0.016)	(0.013)
Particulate Matter	0.316***	0.323***	0.314***
	(0.024)	(0.023)	(0.025)
Maternal/Neonatal Disorders	0.017***	0.014***	0.019***
	(0.003)	(0.003)	(0.003)
Mental Health Policy	-0.002	-0.002	-0.002
	(0.005)	(0.004)	(0.005)
Government Quality	-0.009	-0.008	-0.009
	(0.006)	(0.005)	(0.006)
Out of Pocket expenditures	-0.043*	-0.016	-0.052**
	(0.025)	(0.023)	(0.026)
Population Size	0.370***	0.372***	0.368***
	(0.028)	(0.027)	(0.029)
Sex Ratio at Birth	0.086**	0.109***	0.079**
	(0.038)	(0.035)	(0.039)
Gross Domestic Product	-0.038***	-0.035***	-0.039***
	(0.014)	(0.013)	(0.014)
Year Fixed Effect	Yes	Yes	Yes
Country Fixed Effect	Yes	Yes	Yes
Constant	Yes	Yes	Yes
Observations	2,239	2,239	2,239
R-squared	0.712	0.720	0.707
KPrk LM stat. [p-value]	39.004 [0.000]	39.129 [0.000]	39.004 [0.000]
KPrk Wald F stat.	19.142	23.148	19.142
Hansen-J stat. [p-value]	2.666 [0.103]	0.529 [0.467]	2.529 [0.112]
*First-stage statistics*			
SW Chi-sq [p-value]	38.490 [0.000]	46.540 [0.000]	38.490 [0.000]
SW F-stat	19.140	23.150	19.140

Models estimated via IV-2SLS with fixed-effects (using xtivreg2 in STATA). Kleibergen-Paap rk LM statistic tests for under identification (H₀: instruments irrelevant); a low p-value rejects H₀, supporting instrument relevance. The Kleibergen-Paap rk Wald F statistic tests for weak instruments; an F-statistic > 10 alleviates weak instrument concerns. The Hansen J statistic tests the joint validity of overidentifying restrictions (H₀: all instruments exogenous); a high p-value fails to reject H₀, supporting instrument exogeneity. Sanderson-Windmeijer (SW) F-statistics test for weak instruments in the first-stage regression of each endogenous variable; values > 10 are desired. Standard errors in parentheses ***p<0.01, **p<0.05, *p<0.1.

**Table 10** demonstrates winsorization as a robustness check, capping extreme values to mitigate outlier influence while preserving sample size (145). In order to confirm that our results are not driven by outliers or extreme values, we applied winsorization at 1% and 99% cutoffs. For brevity, the subsample of low and high-income countries were divided by median value of GDP. The analysis confirms our core findings with low GDP countries showing significant positive aid coefficients (0.332***) versus negligible effects (0.015) in high GDP countries. This technique demonstrates that observed patterns reflect genuine structural differences rather than statistical anomalies from extreme observations (36), strengthening our inference about systematic differences across economic development levels.

**Table 10:** Winsorized analysis of aid-autism relationship by GDP Level.

	Countries with low GDP			Countries with high GDP		
	Autism	Females	Males	Autism	Females	Males
Official Aid Received	0.332***	0.341***	0.330***	0.015	0.000	0.020
	(0.037)	(0.039)	(0.037)	(0.037)	(0.035)	(0.038)
Particulate Matter	-0.088***	-0.079**	-0.091***	0.051**	0.065***	0.046**
	(0.030)	(0.031)	(0.030)	(0.022)	(0.021)	(0.023)
Maternal/Neonatal Disorders	0.024**	0.017	0.026**	0.188***	0.166***	0.193***
	(0.010)	(0.010)	(0.010)	(0.012)	(0.011)	(0.012)
Mental Health Policy	0.020	0.024	0.019	0.030	0.020	0.034
	(0.038)	(0.039)	(0.038)	(0.037)	(0.035)	(0.037)
Government Quality	-0.021**	-0.018*	-0.023**	-0.125***	-0.123***	-0.126***
	(0.010)	(0.010)	(0.010)	(0.008)	(0.008)	(0.008)
Out of Pocket expenditures	0.204***	0.218***	0.199***	-0.155***	-0.117***	-0.166***
	(0.055)	(0.057)	(0.055)	(0.046)	(0.044)	(0.047)
Population Size	0.847***	0.841***	0.848***	0.845***	0.853***	0.843***
	(0.012)	(0.013)	(0.012)	(0.007)	(0.006)	(0.007)
Sex Ratio at Birth	-0.449***	-0.546***	-0.416***	-0.624***	-0.766***	-0.579***
	(0.048)	(0.049)	(0.048)	(0.034)	(0.033)	(0.035)
Gross Domestic Product	-0.134***	-0.154***	-0.127***	-0.034**	-0.089***	-0.018
	(0.021)	(0.021)	(0.021)	(0.016)	(0.015)	(0.017)
Year Fixed Effect	YES	YES	YES	YES	YES	YES
Country Fixed effect	YES	YES	YES	YES	YES	YES
Constant	8.330	7.772	7.868	-12.995	-13.546	-13.532
	(9.959)	(10.319)	(9.955)	(9.917)	(9.463)	(10.139)
Observations	752	752	752	1,487	1,487	1,487

Standard errors in parentheses; ***p<0.01, **p<0.05, *p<0.1.

The regression results in **Table 11** reveal valuable insights into the prevalence of autism, mainly highlighting the significance of lag effects. The inclusion of lagged variables in the regression model consents for the examination of the delayed effects of official aid on autism prevalence. This approach is important in understanding the temporal dynamics of the relationship between official assistance and reported cases of autism. Coefficients for L1, L2, and L3 are constantly positive and significant, indicating that an increase in official aid in one year positively influences the prevalence of autism in the following three years. The results of the analysis deliver compelling evidence in favor of the hypothesis, indicating a noteworthy connection between official aid and the subsequent increase in reported autism cases.

**Table 11:** 3-years lagged analysis of official aid received on autism prevalence.

Variables	Autism	Females	Males	Autism	Females	Males	Autism	Females	Males
L1: Autism	0.099***	0.099***	0.100***						
	(0.021)	(0.020)	(0.022)						
L2: Autism				0.106***	0.102***	0.107***			
				(0.022)	(0.021)	(0.022)			
L3: Autism							0.149***	0.136***	0.153***
							(0.025)	(0.024)	(0.026)
Particulate Matter	0.025	0.049***	0.017	0.028	0.050***	0.020	0.029	0.050***	0.022
	(0.018)	(0.017)	(0.019)	(0.018)	(0.017)	(0.019)	(0.019)	(0.018)	(0.019)
MN Disorders	0.051***	0.053***	0.049***	0.052***	0.055***	0.051***	0.053***	0.055***	0.052***
	(0.005)	(0.004)	(0.005)	(0.005)	(0.004)	(0.005)	(0.005)	(0.004)	(0.005)
MH Policy	0.024	0.017	0.026	0.023	0.017	0.026	0.017	0.011	0.019
	(0.031)	(0.029)	(0.031)	(0.031)	(0.029)	(0.032)	(0.031)	(0.030)	(0.032)
GE	-0.103***	-0.097***	-0.105***	-0.106***	-0.100***	-0.109***	-0.110***	-0.103***	-0.112***
	(0.007)	(0.007)	(0.007)	(0.007)	(0.007)	(0.007)	(0.007)	(0.007)	(0.007)
OPE	-0.026	0.006	-0.035	-0.024	0.009	-0.033	-0.022	0.011	-0.032
	(0.038)	(0.036)	(0.039)	(0.039)	(0.037)	(0.039)	(0.039)	(0.037)	(0.040)
Population Size	0.868***	0.871***	0.868***	0.864***	0.868***	0.863***	0.857***	0.862***	0.856***
	(0.006)	(0.005)	(0.006)	(0.006)	(0.005)	(0.006)	(0.006)	(0.005)	(0.006)
Sex Ratio at Birth	-0.658***	-0.765***	-0.623***	-0.663***	-0.770***	-0.628***	-0.664***	-0.770***	-0.628***
	(0.029)	(0.027)	(0.029)	(0.029)	(0.028)	(0.030)	(0.030)	(0.028)	(0.030)
GDP	-0.113***	-0.151***	-0.101***	-0.106***	-0.146***	-0.093***	-0.096***	-0.138***	-0.082***
	(0.009)	(0.009)	(0.010)	(0.010)	(0.009)	(0.010)	(0.010)	(0.009)	(0.010)
Year Fixed Effect	YES	YES	YES	YES	YES	YES	YES	YES	YES
Country Fixed Effect	YES	YES	YES	YES	YES	YES	YES	YES	YES
Constant	10.609***	6.546**	11.106***	10.962***	6.869**	11.463***	11.414***	7.252***	11.932***
	(2.898)	(2.760)	(2.959)	(2.934)	(2.778)	(2.999)	(2.980)	(2.805)	(3.050)
Observations	2,267	2,267	2,267	2,295	2,295	2,295	2,320	2,320	2,320

GE, Government Effectiveness; OPE, Out of Pocket expenditures; MH Policy, Mental Health Policy; MN Disorder, Maternal/Neonatal Disorder; Standard errors in parentheses ***p<0.01, **p<0.01, *p<0.05.

**Figure 6:**
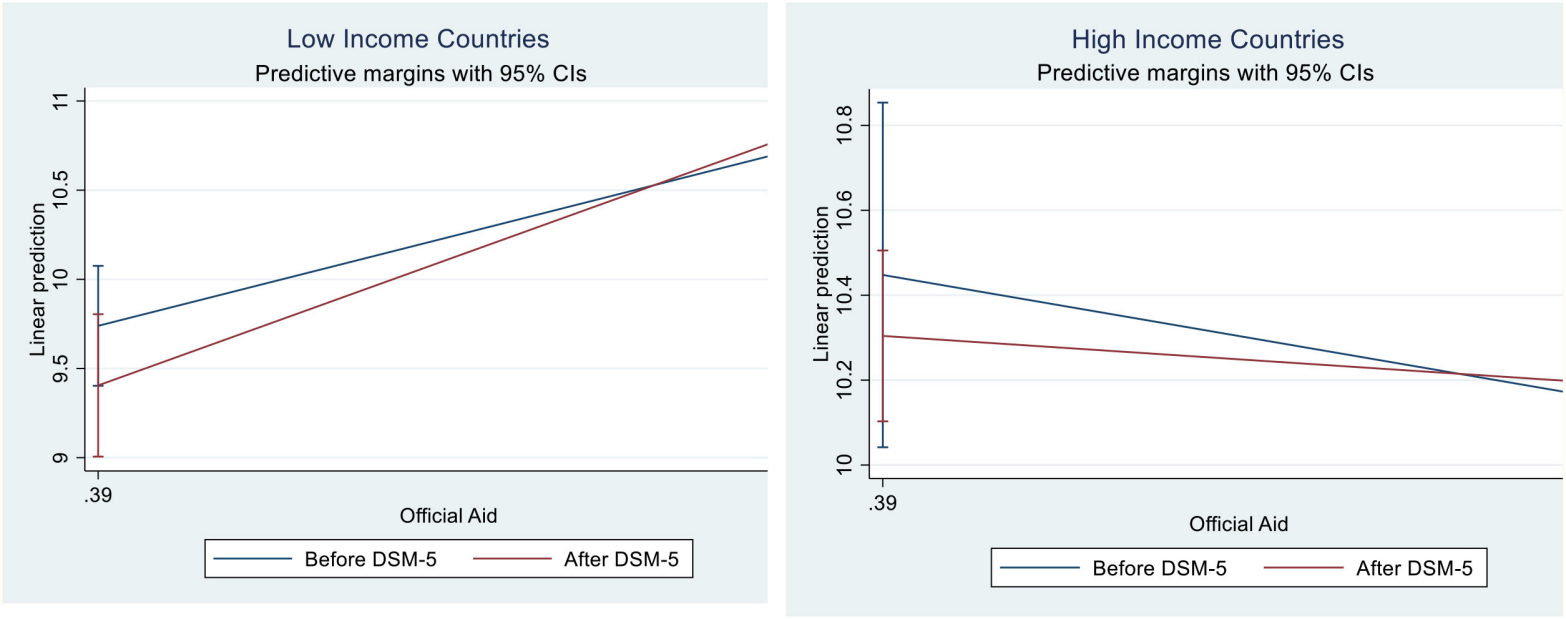
Moderating effect of DSM-5 implementation on the relationship between official aid and ASD prevalence by income level.

The segmented analysis addressing the DSM-5 implementation reveals contrasting patterns in how official aid affects autism prevalence across income groups and diagnostic periods (**Table 12**; **Figure 6**). In low-income countries, official aid demonstrates consistently positive associations with autism prevalence (0.042, p<0.01), with the interaction term (Official Aid*DSM-5) showing significant additional effects ranging from 0.016 to 0.023 (*p* < 0.05 to *p* < 0.01), indicating that the aid-autism relationship intensified following DSM-5 implementation. Conversely, high-income countries exhibit no significant associations between aid and autism prevalence, with negligible interaction effects across all specifications. **Figure 6** illustrates these divergent patterns: low-income countries display sharply steepening slopes post-DSM-5, while high-income countries maintain flat relationships regardless of diagnostic period. These findings present an interpretive challenge, as the observed patterns are consistent with both improved diagnostic capacity in previously under-resourced settings and potential diagnostic inflation enabled by broader DSM-5 criteria and aid-related incentives.

**Table 12:** Interaction effects of DSM-5 implementation on official-aid ASD prevalence relationship by income group.

	Low income countries			High income countries		
VARIABLES	Autism	Females	Males	Autism	Females	Males
Official Aid Received	0.042***	0.044***	0.042***	-0.012	-0.007	-0.014
	(0.008)	(0.008)	(0.008)	(0.010)	(0.008)	(0.011)
DSM-5	-0.341*	-0.454**	-0.294	-0.147	-0.068	-0.171
	(0.189)	(0.185)	(0.191)	(0.211)	(0.174)	(0.226)
Official Aid* DSM-5	0.018**	0.023***	0.016*	0.008	0.004	0.009
	(0.009)	(0.009)	(0.009)	(0.010)	(0.008)	(0.011)
Particulate Matter	0.208***	0.225***	0.203***	0.128***	0.125***	0.130***
	(0.020)	(0.020)	(0.020)	(0.020)	(0.016)	(0.021)
Maternal & Neonatal Disorder	0.030***	0.028***	0.031***	0.106***	0.061***	0.118***
	(0.005)	(0.005)	(0.005)	(0.025)	(0.020)	(0.026)
Mental Health Policy	-0.006	-0.006	-0.005	0.000	0.002	-0.000
	(0.007)	(0.007)	(0.007)	(0.005)	(0.004)	(0.006)
Government Effectiveness	-0.020***	-0.021***	-0.020***	-0.022***	-0.014***	-0.024***
	(0.005)	(0.005)	(0.005)	(0.005)	(0.004)	(0.006)
Out of Pocket expenditures	-0.132***	-0.113***	-0.140***	-0.162***	-0.106***	-0.178***
	(0.022)	(0.022)	(0.022)	(0.022)	(0.018)	(0.024)
Population Size	0.532***	0.526***	0.533***	0.331***	0.322***	0.334***
	(0.018)	(0.018)	(0.018)	(0.019)	(0.016)	(0.020)
Sex Ratio at Birth	0.190***	0.182***	0.192***	0.003	0.110***	-0.028
	(0.034)	(0.033)	(0.034)	(0.033)	(0.028)	(0.036)
GDP	-0.014	-0.014	-0.014	-0.057***	-0.049***	-0.059***
	(0.011)	(0.011)	(0.011)	(0.014)	(0.012)	(0.015)
Constant	YES	YES	YES	YES	YES	YES
	YES	YES	YES	YES	YES	YES
Observations	1,439	1,439	1,439	800	800	800
R-squared	0.771	0.776	0.768	0.655	0.705	0.635

Standard errors in parentheses ***p<0.01, **p<0.01, *p<0.05. The subsample is divided based on the median value of GDP. DSM-5 indicates dummy variable, i.e. 1 for the years after the introduction of DSM-5 (2013), 0 for the years before 2012.

Summary. The pronounced positive relationship between official aid and autism prevalence in low-income countries with limited healthcare infrastructure suggests a complex interplay of legitimate diagnostic improvements and potential systematic biases. In these under-resourced settings, aid likely enables genuine case identification by providing access to specialized diagnostic services, training healthcare providers in autism recognition, and establishing screening programs that previously did not exist, thereby capturing a substantial population of undiagnosed individuals who lacked access to appropriate evaluation. However, the intensification of this relationship following DSM-5 implementation, particularly in countries with weak governance structures and limited mental health oversight, indicates that expanded diagnostic criteria may have created conditions conducive to both improved detection and diagnostic inflation operating simultaneously. The contrast with high-income countries, where established healthcare systems and robust oversight mechanisms prevent such pronounced aid-diagnosis correlations, suggests that institutional quality plays a crucial role in determining whether increased resources translate into accurate diagnostic improvements or systematic over-reporting. These findings underscore that while aid can meaningfully enhance autism identification capacity in previously neglected populations, the absence of adequate regulatory frameworks and professional oversight may allow legitimate diagnostic expansion to drift toward systematic inflation, necessitating careful consideration of both the beneficial and potentially distortive effects of targeted healthcare funding.

## 5 Clinical and policy implications

The findings of this research carry profound implications for both policymakers and clinical practitioners operating within the global health architecture. If the overdiagnosis hypothesis is confirmed, international donors and recipient governments must fundamentally reconsider aid allocation mechanisms to prevent perverse diagnostic incentives that distort epidemiological surveillance systems. Policymakers should implement robust oversight frameworks that decouple healthcare funding from reported disease prevalence, instead tying aid disbursements to process indicators such as healthcare infrastructure development, provider training quality, and diagnostic protocol adherence. Clinical practitioners in aid-recipient countries require enhanced training not only in accurate autism identification but also in resisting institutional pressures that may encourage diagnostic inflation, necessitating the establishment of independent clinical review boards and peer consultation networks that can validate complex neurodevelopmental diagnoses.

Conversely, if the relationship reflects genuine diagnostic improvement, the implications suggest that targeted aid interventions can effectively enhance autism detection capacity in under-resourced settings, warranting continued investment in specialized healthcare infrastructure and provider education. However, even under this interpretation, the dramatic differences observed across governance contexts indicate that aid effectiveness is highly conditional on institutional quality, suggesting that donors should prioritize governance strengthening alongside healthcare capacity building. For clinical practice, these findings underscore the critical importance of implementing standardized diagnostic protocols and ensuring adequate specialist supervision, particularly during periods of rapid healthcare system expansion. Regardless of the underlying mechanism, the research demonstrates an urgent need for enhanced data verification systems, independent diagnostic auditing processes, and transparent reporting mechanisms that can distinguish between authentic epidemiological trends and artifact-driven prevalence increases, ensuring that autism prevalence data serves as a reliable foundation for evidence-based policy and clinical decision-making.

## 6 Limitations

Several methodological constraints limit definitive causal interpretation beyond the interpretive ambiguity already discussed. The reliance on IHME data introduces significant constraints, as these estimates assume relatively uniform diagnostic criteria application globally, which may not reflect substantial heterogeneity in clinical practice, healthcare infrastructure, and cultural understanding of autism across diverse national contexts. The Benford’s Law findings themselves may be influenced by variations in local diagnostic practices, cultural interpretations of autism symptoms, and country-specific reporting protocols that could generate digit irregularities independent of deliberate data manipulation. The analysis lacks granular data on diagnostic severity distributions, healthcare provider incentives, and specific aid conditionality mechanisms that would enable clearer mechanistic understanding. Furthermore, the study cannot account for country-specific variations in DSM-5 implementation timing, training quality, or institutional oversight that may influence diagnostic practices. The temporal aggregation of data may also mask important within-period variations in both aid flows and diagnostic practices that could provide additional insights into underlying causal mechanisms.

## 7 Conclusion

The relationship between international aid and autism spectrum disorder (ASD) prevalence presents a paradoxical empirical puzzle that challenges conventional assumptions about development assistance effectiveness in health outcomes. This research reveals a substantial positive association between official aid received by countries worldwide and subsequent ASD incidence, yet this relationship embodies complex interpretive challenges that extend beyond simple causality. The study employed rigorous statistical controls, including instrumental variable analysis establishing causal validation, to address potential confounding variables and endogeneity concerns, thereby enhancing result robustness. The segmented analysis examining DSM-5 implementation (2013) reveals particularly pronounced patterns whereby the aid-autism relationship intensified in low-income countries following broader diagnostic criteria introduction, while high-income countries demonstrated no significant associations. This divergence raises critical questions regarding whether observed increases reflect genuine diagnostic improvements in previously under-resourced settings or constitute potential diagnostic inflation facilitated by expanded criteria and aid-related incentives. The examination of potential ASD data misreporting across income groups employed Median Absolute Deviation analysis, revealing significant variability in low-income countries, while Benford’s Law application provided compelling statistical evidence of irregularities that corroborate systematic distortion concerns in autism reporting, particularly within aid-dependent settings.

The study’s nuanced data segregation reveals contextual variations in aid-autism relationships, with countries possessing limited mental health services demonstrating distinct positive correlations with official aid, whereas this association became non-significant in nations with well-established mental health services. The observed patterns suggest that while aid may enhance diagnostic capacity in under-resourced settings, it may simultaneously create conditions conducive to systematic over-reporting, necessitating cautious policy formulation given the interpretive ambiguity between diagnostic improvement and potential overdiagnosis. International health organizations should prioritize both data integrity in ASD reporting and robust oversight mechanism development that distinguishes between genuine case detection and diagnostic inflation. Enhanced data accuracy and verification systems prove crucial for informed decision-making and resource allocation, ensuring interventions address authentic needs rather than artificially inflated prevalence figures. This research emphasizes the critical importance of maintaining vigilance in health data quality monitoring while acknowledging that aid-autism prevalence relationships reflect complex institutional, economic, and diagnostic factors requiring further investigation to definitively resolve underlying causal mechanisms.

## Data Availability

The datasets presented in this study can be found in online repositories. The names of the repository/repositories and accession number(s) can be found in the article/**Supplementary Material**.
