# Supplementary material for: Red flags in global autism data: a forensic analysis of prevalence patterns and official aid dependencies
Source: Front Psychiatry. 2025 Oct 15;16:1575940. doi: 10.3389/fpsyt.2025.1575940 (PMC12569649; doi:10.3389/fpsyt.2025.1575940)
Supplement: Supplementary file 1 [file Table1.docx]

**STATA-COMMANDS**

**Main Results**

reg lnAutismTotal lnAidRec1 lnPmatter NND1 mhpolicy GQ1 lnOPEx1 lnPOPS2 lnSEX lnGDP1

reg lnAutismTotal lnAidRec1 lnPmatter NND1 mhpolicy GQ1 lnOPEx1 lnPOPS2 lnSEX lnGDP1 year* IDgroup

reg lnMaleAutism1 lnAidRec1 lnPmatter NND1 mhpolicy GQ1 lnOPEx1 lnPOPS2 lnSEX lnGDP1 year* IDgroup

reg lnFemaleAutism1 lnAidRec1 lnPmatter NND1 mhpolicy GQ1 lnOPEx1 lnPOPS2 lnSEX lnGDP1 year* IDgroup

**Segregation**

**MHS**

reg lnAutismTotal lnAidRec1 lnPmatter NND1 mhpolicy GQ1 lnOPEx1 lnPOPS2 lnSEX lnGDP1 year* IDgroup if mhs <8.03

reg lnFemaleAutism1 lnAidRec1 lnPmatter NND1 mhpolicy GQ1 lnOPEx1 lnPOPS2 lnSEX lnGDP1 year* IDgroup if mhs <8.03

reg lnMaleAutism1 lnAidRec1 lnPmatter NND1 mhpolicy GQ1 lnOPEx1 lnPOPS2 lnSEX lnGDP1 year* IDgroup if mhs <8.03

reg lnAutismTotal lnAidRec1 lnPmatter NND1 mhpolicy GQ1 lnOPEx1 lnPOPS2 lnSEX lnGDP1 year* IDgroup if mhs >=8.03

reg lnFemaleAutism1 lnAidRec1 lnPmatter NND1 mhpolicy GQ1 lnOPEx1 lnPOPS2 lnSEX lnGDP1 year* IDgroup if mhs >=8.03

reg lnMaleAutism1 lnAidRec1 lnPmatter NND1 mhpolicy GQ1 lnOPEx1 lnPOPS2 lnSEX lnGDP1 year* IDgroup if mhs >=8.03

**Income Category**

reg lnAutismTotal lnAidRec1 lnPmatter NND1 mhpolicy GQ1 lnOPEx1 lnPOPS2 lnSEX lnGDP1 year* IDgroup if income== 1

reg lnMaleAutism1 lnAidRec1 lnPmatter NND1 mhpolicy GQ1 lnOPEx1 lnPOPS2 lnSEX lnGDP1 year* IDgroup if income== 1

reg lnFemaleAutism1 lnAidRec1 lnPmatter NND1 mhpolicy GQ1 lnOPEx1 lnPOPS2 lnSEX lnGDP1 year* IDgroup if income== 1

reg lnAutismTotal lnAidRec1 lnPmatter NND1 mhpolicy GQ1 lnOPEx1 lnPOPS2 lnSEX lnGDP1 year* IDgroup if income== 2

reg lnMaleAutism1 lnAidRec1 lnPmatter NND1 mhpolicy GQ1 lnOPEx1 lnPOPS2 lnSEX lnGDP1 year* IDgroup if income== 2

reg lnFemaleAutism1 lnAidRec1 lnPmatter NND1 mhpolicy GQ1 lnOPEx1 lnPOPS2 lnSEX lnGDP1 year* IDgroup if income== 2

reg lnAutismTotal lnAidRec1 lnPmatter NND1 mhpolicy GQ1 lnOPEx1 lnPOPS2 lnSEX lnGDP1 year* IDgroup if income== 3

reg lnMaleAutism1 lnAidRec1 lnPmatter NND1 mhpolicy GQ1 lnOPEx1 lnPOPS2 lnSEX lnGDP1 year* IDgroup if income== 3

reg lnFemaleAutism1 lnAidRec1 lnPmatter NND1 mhpolicy GQ1 lnOPEx1 lnPOPS2 lnSEX lnGDP1 year* IDgroup if income== 3

reg lnAutismTotal lnAidRec1 lnPmatter NND1 mhpolicy GQ1 lnOPEx1 lnPOPS2 lnSEX lnGDP1 year* IDgroup if income== 4

reg lnMaleAutism1 lnAidRec1 lnPmatter NND1 mhpolicy GQ1 lnOPEx1 lnPOPS2 lnSEX lnGDP1 year* IDgroup if income== 4

reg lnFemaleAutism1 lnAidRec1 lnPmatter NND1 mhpolicy GQ1 lnOPEx1 lnPOPS2 lnSEX lnGDP1 year* IDgroup if income== 4

| 1 | HIC |
| --- | --- |
| 2 | LIC |
| 3 | LMC |
| 4 | UMC |

**Health Expenditure**

*Current health expenditure per capita (current US$)*

|  | Percentiles |
| --- | --- |
| 1% | 8.684352 |
| 5% | 18.62379 |
| 10% | 26.5111 |
| 25% | 61.78501 |
| 50% | 250.3946 |
| 75% | 826.2829 |
| 90% | 2895.807 |
| 95% | 4644.596 |
| 99% | 7297.38 |

**Low HE**

reg lnAutismTotal lnAidRec1 lnPmatter NND1 mhpolicy GQ1 lnOPEx1 lnPOPS2 lnSEX lnGDP1 year* IDgroup if HExp <61.785

reg lnFemaleAutism1 lnAidRec1 lnPmatter NND1 mhpolicy GQ1 lnOPEx1 lnPOPS2 lnSEX lnGDP1 year* IDgroup if HExp <61.785

reg lnMaleAutism1 lnAidRec1 lnPmatter NND1 mhpolicy GQ1 lnOPEx1 lnPOPS2 lnSEX lnGDP1 year* IDgroup if HExp <61.785

Medium HE

reg lnAutismTotal lnAidRec1 lnPmatter NND1 mhpolicy GQ1 lnOPEx1 lnPOPS2 lnSEX lnGDP1 year* IDgroup if HExp >=61.79 & HExp <= 826.28

reg lnFemaleAutism1 lnAidRec1 lnPmatter NND1 mhpolicy GQ1 lnOPEx1 lnPOPS2 lnSEX lnGDP1 year* IDgroup if HExp >=61.79 & HExp <= 826.28

reg lnMaleAutism1 lnAidRec1 lnPmatter NND1 mhpolicy GQ1 lnOPEx1 lnPOPS2 lnSEX lnGDP1 year* IDgroup if HExp >=61.79 & HExp <= 826.28

HHE

reg lnAutismTotal lnAidRec1 lnPmatter NND1 mhpolicy GQ1 lnOPEx1 lnPOPS2 lnSEX lnGDP1 year* IDgroup if >826.28

reg lnFemaleAutism1 lnAidRec1 lnPmatter NND1 mhpolicy GQ1 lnOPEx1 lnPOPS2 lnSEX lnGDP1 year* IDgroup if HExp >826.28

reg lnMaleAutism1 lnAidRec1 lnPmatter NND1 mhpolicy GQ1 lnOPEx1 lnPOPS2 lnSEX lnGDP1 year* IDgroup if HExp >826.28

**GDP**

| Percentiles |
| --- |
| 1% 292.1294 |
| 5% 460.6085 |
| 10% 633.9426 |
| 25% 1475.355 |
| 50% 4255.863 |
| 75% 14664.24 |
| 90% 37938.26 |
| 95% 49903.9 |
| 99% 105339 |

Low GDP

reg lnAutismTotal lnAidRec1 lnPmatter NND1 mhpolicy GQ1 lnOPEx1 lnPOPS2 lnSEX lnGDP1 year* IDgroup if GDP <1475.35

reg lnFemaleAutism1 lnAidRec1 lnPmatter NND1 mhpolicy GQ1 lnOPEx1 lnPOPS2 lnSEX lnGDP1 year* IDgroup if GDP <1475.35

reg lnMaleAutism1 lnAidRec1 lnPmatter NND1 mhpolicy GQ1 lnOPEx1 lnPOPS2 lnSEX lnGDP1 year* IDgroup if GDP <1475.35

Medium GDP

reg lnAutismTotal lnAidRec1 lnPmatter NND1 mhpolicy GQ1 lnOPEx1 lnPOPS2 lnSEX lnGDP1 year* IDgroup if GDP >=1475.35 & GDP <=14664.24

reg lnFemaleAutism1 lnAidRec1 lnPmatter NND1 mhpolicy GQ1 lnOPEx1 lnPOPS2 lnSEX lnGDP1 year* IDgroup if GDP >=1475.35 & GDP <=14664.24

reg lnMaleAutism1 lnAidRec1 lnPmatter NND1 mhpolicy GQ1 lnOPEx1 lnPOPS2 lnSEX lnGDP1 year* IDgroup if GDP >=1475.35 & GDP <=14664.24

**High GDP**

reg lnAutismTotal lnAidRec1 lnPmatter NND1 mhpolicy GQ1 lnOPEx1 lnPOPS2 lnSEX lnGDP1 year* IDgroup if GDP >14664.24

reg lnFemaleAutism1 lnAidRec1 lnPmatter NND1 mhpolicy GQ1 lnOPEx1 lnPOPS2 lnSEX lnGDP1 year* IDgroup if GDP >14664.24

reg lnMaleAutism1 lnAidRec1 lnPmatter NND1 mhpolicy GQ1 lnOPEx1 lnPOPS2 lnSEX lnGDP1 year* IDgroup if GDP >14664.24

**Government Effectiveness**

| Percentiles |
| --- |
| 1% -1.928121 |
| 5% -1.494259 |
| 10% -1.207594 |
| 25% -.7778762 |
| 50% -.1948338 |
| 75% .5880329 |
| 90% 1.467408 |
| 95% 1.782482 |
| 99% 2.0705 |

Low GE

reg lnAutismTotal lnAidRec1 lnPmatter NND1 mhpolicy GQ1 lnOPEx1 lnPOPS2 lnSEX lnGDP1 year* IDgroup if GovtEff <-.778

reg lnFemaleAutism1 lnAidRec1 lnPmatter NND1 mhpolicy GQ1 lnOPEx1 lnPOPS2 lnSEX lnGDP1 year* IDgroup if GovtEff <-.778

reg lnMaleAutism1 lnAidRec1 lnPmatter NND1 mhpolicy GQ1 lnOPEx1 lnPOPS2 lnSEX lnGDP1 year* IDgroup if GovtEff <-.778

Medium GE

reg lnAutismTotal lnAidRec1 lnPmatter NND1 mhpolicy GQ1 lnOPEx1 lnPOPS2 lnSEX lnGDP1 year* IDgroup if GovtEff >=-.778 & GovtEff <=.588

reg lnFemaleAutism1 lnAidRec1 lnPmatter NND1 mhpolicy GQ1 lnOPEx1 lnPOPS2 lnSEX lnGDP1 year* IDgroup if GovtEff <=-.778 & GovtEff<=.588

reg lnMaleAutism1 lnAidRec1 lnPmatter NND1 mhpolicy GQ1 lnOPEx1 lnPOPS2 lnSEX lnGDP1 year* IDgroup if GovtEff <=-.778 & GovtEff<=.588

High GE

reg lnAutismTotal lnAidRec1 lnPmatter NND1 mhpolicy GQ1 lnOPEx1 lnPOPS2 lnSEX lnGDP1 year* IDgroup if GovtEff>.588

reg lnFemaleAutism1 lnAidRec1 lnPmatter NND1 mhpolicy GQ1 lnOPEx1 lnPOPS2 lnSEX lnGDP1 year* IDgroup if GovtEff >.588

reg lnMaleAutism1 lnAidRec1 lnPmatter NND1 mhpolicy GQ1 lnOPEx1 lnPOPS2 lnSEX lnGDP1 year* IDgroup if GovtEff >.588

**Economist Democracy Index**

1= Full democracies

2= Flawed democracies

3= Hybrid regimes

4= Authoritarian regimes

reg lnAutismTotal lnAidRec1 lnPmatter NND1 mhpolicy GQ1 lnOPEx1 lnPOPS2 lnSEX lnGDP1 year* IDgroup if Regime == 1

reg lnMaleAutism1 lnAidRec1 lnPmatter NND1 mhpolicy GQ1 lnOPEx1 lnPOPS2 lnSEX lnGDP1 year* IDgroup if Regime == 1

reg lnFemaleAutism1 lnAidRec1 lnPmatter NND1 mhpolicy GQ1 lnOPEx1 lnPOPS2 lnSEX lnGDP1 year* IDgroup if Regime == 1

reg lnAutismTotal lnAidRec1 lnPmatter NND1 mhpolicy GQ1 lnOPEx1 lnPOPS2 lnSEX lnGDP1 year* IDgroup if Regime == 2

reg lnMaleAutism1 lnAidRec1 lnPmatter NND1 mhpolicy GQ1 lnOPEx1 lnPOPS2 lnSEX lnGDP1 year* IDgroup if Regime == 2

reg lnFemaleAutism1 lnAidRec1 lnPmatter NND1 mhpolicy GQ1 lnOPEx1 lnPOPS2 lnSEX lnGDP1 year* IDgroup if Regime == 2

reg lnAutismTotal lnAidRec1 lnPmatter NND1 mhpolicy GQ1 lnOPEx1 lnPOPS2 lnSEX lnGDP1 year* IDgroup if Regime == 2

reg lnMaleAutism1 lnAidRec1 lnPmatter NND1 mhpolicy GQ1 lnOPEx1 lnPOPS2 lnSEX lnGDP1 year* IDgroup if Regime == 2

reg lnFemaleAutism1 lnAidRec1 lnPmatter NND1 mhpolicy GQ1 lnOPEx1 lnPOPS2 lnSEX lnGDP1 year* IDgroup if Regime == 2

reg lnAutismTotal lnAidRec1 lnPmatter NND1 mhpolicy GQ1 lnOPEx1 lnPOPS2 lnSEX lnGDP1 year* IDgroup if Regime == 3

reg lnMaleAutism1 lnAidRec1 lnPmatter NND1 mhpolicy GQ1 lnOPEx1 lnPOPS2 lnSEX lnGDP1 year* IDgroup if Regime == 3

reg lnFemaleAutism1 lnAidRec1 lnPmatter NND1 mhpolicy GQ1 lnOPEx1 lnPOPS2 lnSEX lnGDP1 year* IDgroup if Regime == 3

reg lnAutismTotal lnAidRec1 lnPmatter NND1 mhpolicy GQ1 lnOPEx1 lnPOPS2 lnSEX lnGDP1 year* IDgroup if Regime == 4

reg lnMaleAutism1 lnAidRec1 lnPmatter NND1 mhpolicy GQ1 lnOPEx1 lnPOPS2 lnSEX lnGDP1 year* IDgroup if Regime == 4

reg lnFemaleAutism1 lnAidRec1 lnPmatter NND1 mhpolicy GQ1 lnOPEx1 lnPOPS2 lnSEX lnGDP1 year* IDgroup if Regime == 4

**3 Year Lagged Regression**

reg lnAutismTotal l.lnAidRec1 lnPmatter NND1 mhpolicy GQ1 lnOPEx1 lnPOPS2 lnSEX lnGDP1 year* IDgroup

reg lnFemaleAutism1 l.lnAidRec1 lnPmatter NND1 mhpolicy GQ1 lnOPEx1 lnPOPS2 lnSEX lnGDP1 year* IDgroup

reg lnMaleAutism1 l.lnAidRec1 lnPmatter NND1 mhpolicy GQ1 lnOPEx1 lnPOPS2 lnSEX lnGDP1 year* IDgroup

reg lnAutismTotal l2.lnAidRec1 lnPmatter NND1 mhpolicy GQ1 lnOPEx1 lnPOPS2 lnSEX lnGDP1 year* IDgroup

reg lnFemaleAutism1 l2.lnAidRec1 lnPmatter NND1 mhpolicy GQ1 lnOPEx1 lnPOPS2 lnSEX lnGDP1 year* IDgroup

reg lnMaleAutism1 l2.lnAidRec1 lnPmatter NND1 mhpolicy GQ1 lnOPEx1 lnPOPS2 lnSEX lnGDP1 year* IDgroup

reg lnAutismTotal l3.lnAidRec1 lnPmatter NND1 mhpolicy GQ1 lnOPEx1 lnPOPS2 lnSEX lnGDP1 year* IDgroup

reg lnFemaleAutism1 l3.lnAidRec1 lnPmatter NND1 mhpolicy GQ1 lnOPEx1 lnPOPS2 lnSEX lnGDP1 year* IDgroup

reg lnMaleAutism1 l3.lnAidRec1 lnPmatter NND1 mhpolicy GQ1 lnOPEx1 lnPOPS2 lnSEX lnGDP1 year* IDgroup
